# Investigation of serum thyroid hormones, iodine and cobalt concentrations across common aquarium-housed elasmobranchs

**DOI:** 10.3389/fvets.2025.1504527

**Published:** 2025-02-26

**Authors:** Catharine J. Wheaton, Kathleen E. Sullivan, Enass Bassiouny, Charlene M. Burns, Matthew J. Smukall, Jill M. Hendon, Natalie D. Mylniczenko

**Affiliations:** ^1^Disney’s Animals, Science and Environment, Animal Programs, Lake Buena Vista, FL, United States; ^2^Michigan State University Veterinary Diagnostic Laboratory, College of Veterinary Medicine, East Lansing, MI, United States; ^3^Bimini Biological Field Station, South Bimini, Bahamas; ^4^The University of Southern Mississippi, Center for Fisheries Research and Development, Ocean Springs, MS, United States

**Keywords:** Total T3, Total T4, free T4, triiodothyronine (T3), tetraiodothyronine (thyroxine; T4), reproductive disease, ultrasound, thyroid disease

## Abstract

**Introduction:**

Thyroid disease is an important condition to understand in elasmobranchs, with goiters being predominant. To identify dysfunction, measuring serum thyroid hormone levels is a standard of practice for diagnosing disease in most species. Although these levels have been reported in elasmobranch literature, the testing methodology is varied and values are not clinically useful for most aquarium species. In a group of aquarium-housed elasmobranchs, thyroid hormone levels had been persistently low or not detectable in otherwise healthy animals as well as animals with thyroid disease. The concern for reliability of these results to diagnose thyroid disease, prompted a shift to serum iodine levels as a proxy to determine thyroid health.

**Methods:**

This study assesses thyroid hormone and iodine levels as compared to thyroid disease stage in elasmobranchs with and without dietary supplementation, to determine the efficacy of using these serum values to guide clinical decisions.

**Results:**

Serum thyroid hormone results were lower than the readable range of the standard curve in both sharks and rays; thus reported values are usually extrapolated. Including additional standards down to the limit of sensitivity improved detection, however increasing the sample volume tested was determined to be the most important factor for obtaining measurable results in low-value thyroid hormone samples. Serum iodine levels are reported in three groups of southern stingrays (*Hypanus americanus*). Other elasmobranch species maintained in aquaria with and without thyroid disease were used for biological comparisons. Non-goiter, diseased animals reliably had elevated levels (over baseline) of thyroid hormones and iodine; in goiter cases, hormones were not useful. Additionally, it was found that cobalt levels were also elevated in some disease states and correlated positively with serum iodine levels.

**Conclusion:**

Current available thyroid testing may not provide clinically useful values unless methodology is adjusted, or disease is severe. Serum iodine may be a useful marker to investigate thyroid health. Further, while thyroid disease may be identifiable with thyroid hormones, it is not straightforward or substantial enough alone for diagnosis.

## 1 Introduction

The thyroid gland plays a critical role for fish maturation, reproduction and osmoregulation (1–5). Due to reproductive diseases in elasmobranchs in aquaria (6), investigation into thyroid activity is important as it has been shown that thyroid and reproductive activity parallel each other (1, 4, 5, 7). However, results from the cohorts reported here (from send-out laboratory tests) routinely showed low or undetectable thyroid levels, where ‘normal’ values were expected (compared with other species ranges, particularly mammals) so it was of interest to determine if normal values were truly low in concentration.

Thyroid disease in elasmobranchs is most commonly associated with goiters (hyperplastic, diffuse colloid or multinodular goiter) but thyroiditis and thyroid carcinoma have also been reported (8, 9). Overall thyroid disease is poorly represented in the elasmobranch pathology literature and this is likely, in part, because the thyroid is frequently not included in the submitted set of tissues (10). Subclinical conditions in the thyroid seem plausible. Additionally, ultrasonographic examination by the authors during diagnostic examinations often showed abnormal thyroids (with multifocal cysts or mineralizations) that did not classify as classic hyperplastic or colloidal goiters (unpublished data).

In the absence of a reliable or validated testing method for thyroids, the use of serum iodine was investigated for elasmobranchs under human care (The Seas with Nemo and Friends®, Lake Buena Vista, Florida, United States) as it has been shown to be correlated with thyroid health in other animals (11–13). Iodine’s sole role in the vertebrate is in thyroid hormone synthesis (14, 15) and iodine levels are correlated with thyroid levels and ultimately thyroid disease (16–18), therefore it was expected that iodine values could translate into a meaningful test for elasmobranch thyroid health. Using iodine assays is useful because it does not require species-specific validation that is required with antibody focused assays, does not require sample treatment (like extraction) and there is no interference with sample components (19–21). Initial values in southern stingrays (*Hypanus americanus*) showed some surprisingly high iodine values as compared to mammalian ranges (19–21) and to ranges from other elasmobranchs in the facility and in the wild (22, 23). These rays were in moderate stages of reproductive disease (22, 23). This suggested further investigation and once again revitalized the need to understand thyroid hormones in elasmobranchs, particularly in regard to iodine and how it related to abnormal thyroids as evaluated by high serum iodine and identification of abnormal thyroid glands on ultrasound or postmortem examination.

This paper investigates serum iodine values in two populations of ‘thyroid-normal’ southern stingrays in natural sea water and compares it to validated thyroid hormone levels in an aquarium-housed population. Additionally, several elasmobranchs with goiters and other thyroid pathology were evaluated and compared to normal animals as a biological validation. Parallel to iodine testing, cobalt (through trace nutrient analysis) was additionally identified with levels above expectation, therefore, was included in this analysis.

## 2 Materials and methods

This is a retrospective study using banked samples and available ultrasound images through routine examinations or health assessments.

### 2.1 Animals

#### 2.1.1 Study 1

Southern stingrays (SR) from three habitats (see Supplementary Table S1 for additional details): aquarium-housed (*N* = 38, closed system), lagoon-housed (*N* = 22, managed, semi-wild), and wild (*N* = 11). For all stingrays, disk width was obtained ventrally by measuring the span between the tips of the widest portion of the pectoral fins. Housing, restraint, and sample collection is described elsewhere (6). For lagoon-housed animals, samples were collected prior to 2019 under a permit to conduct scientific research (No. MAF/LIA/22) from the Department of Fisheries of the Commonwealth of the Bahamas. Wild animals included 11 wild adult females from Bimini, Bahamas, collected under protocols from the Bimini Biological Field Station Foundation, and were released after sampling. Sampling was limited to the spring in both cohorts therefore seasonality could not be assessed.

#### 2.1.2 Study 2

Eight aquarium-managed elasmobranch species were included in this cohort [brownbanded bamboo shark (BBBS); cownose ray (CNR); honeycomb ray (HCR); porcupine ray (PR); round ribbontail ray (RRR); spotted eagle ray (SER); southern stingray (SR); and whitespotted bamboo shark (WSBS); see Supplementary Table S1 for size, sex, scientific name, sample collection years and species N]. Banked samples were originally collected during routine examinations under anesthesia (with varied doses and protocols) and included ultrasound evaluations of the thyroid when possible.

Animals were considered to be in normal health based on physical exam and in-house blood work ranges for complete blood count and serum chemistry. The only variation was an obvious thyroid abnormality as evaluated by ultrasound and subsequently categorized. Post-mortem sample: thyroid tissue and blood was opportunistically collected post-mortem from an SR (*N* = 1) caught in a longline survey through The University of Southern Mississippi, Ocean Springs, MS. Thyroid tissue (extracted for HPLC fractioning) and matching cardiac blood was used for initial thyroid assay (TT3, TT4, FT4) biological validations.

### 2.2 Nutrition

Aquarium-housed elasmobranchs were fed a variety of fish and shellfish (capelin, clam, shrimp, herring, and smelt) based on body weight. Animals were offered an elasmobranch micronutrient supplement (hereafter termed ‘supplement’) specifically made for Disney’s Animal Kingdom (Table 1) at the recommended dose [1 tablet (1.5 g) per ½ lb. of fish; Mazuri Vita-Zu Shark/Ray tabs 5M1Y, Mazuri Exotic Animal Nutrition, St. Louis, MO]. This supplement was altered from the commercial product (5MD8-2011 analysis shown) during the course of this data collection at Disney’s request unrelated to this study at the time (Table 1). Supplements were hidden inside target-fed fish, but consumption was variable across species and individuals, and not always able to be clearly tracked. SRs in the aquarium setting had vitamins withheld from 8/1/2015 to 9/28/2016 as a response to elevated levels of iodine.

**Table 1:** Analysis of percent dry matter, cobalt (ppm), and iodine (ppm) levels in a commercially available shark/ray micronutrient supplement tablet (assigned a letter supplement code based on batch analyzed) and aquatic gel, as well as average levels for commonly fed diet items in the aquarium setting, Atlantic capelin, Norwegian herring, cherrystone clams, lake smelt, white shrimp, and squid without heads or pens.

Supplement type	Date of analysis	Dry matter (%)	Cobalt (ppm DM)	Iodine (ppm DM)	Supplement code N (%)^a^
Shark/ray micronutrient supplement tablet (5M1Y; 5MD8)^b^	5/5/2011^b^	86.8	151.4	191,819.9	“A” 10 (5.6)
	10/3/2012	92.6	4.2	202,603.4	“B” 8 (4.5)
	3/9/2015	93.1	3.8	133,658.3	“C” 32 (17.9)
	8/10/2022	83.7	1.4	104,714.6	“D” 66 (36.9)
	1/31/2023	98.6	0.3	51,043.8	“E” 4 (2.2)
	various	N/A	N/A	N/A	“N” 34 (19.0)
Aquatic gel 57W9^b^	4/17/2016	91.4	3.5	2.12^c^	“G” 25 (14.0)
Atlantic capelin^d^	various	18.5	0.004	1.2	N/A
Norwegian herring^d^	various	30.4	0.01	0.1	N/A
Cherrystone clam^d^	various	78.7	2.7	0.9	N/A
Lake smelt^d^	various	19.2	0.03	0.1	N/A
White shrimp^d^	various	21.3	0.4	2.3	N/A
Squid without heads or pen^d^	various	81.8	0.1	0.1	N/A

a Number and (%) of total study samples for that micronutrient supplement product. Products are labeled as A, B, C, D, E, N, or G to identify the batch used based on date.

b 2011 sample is 5MD8 and all others are 5M1Y tablets, except aquatic gel 57 W9; all products: Mazuri Exotic Animal Nutrition, St. Louis, MO analyzed at Dairy One Laboratories, Ithaca, NY.

c Estimate from manufacturer, product specifications accurate to 2016.

d All analyses performed at Dairy One Laboratory within last 10 years of 2024; for average values; capelin, *N* = 26; clam, *N* = 3; shrimp, *N* = 4; herring, *N* = 40; lake smelt, *N* = 11; squid, *N* = 3.

Lagoon animals were housed in a netted lagoon pen in natural sea water (Castaway Cay, Bahamas). They were fed a daily diet consisting of shrimp with shells but no tails (20% total diet), squid without heads or pens (30% total diet), and an aquatic gel product fed as 50% of the total diet offered (Aquatic gel 57 W9, Mazuri Exotic Animal Nutrition, St Louis, MO).

Wild SR diets were unknown, but diet and stable isotope analysis has been described (24).

### 2.3 Thyroid hormone analysis: biological and chemical validations

#### 2.3.1 Reagents

All reagents used were LC/MS or HPLC analytical grade quality. Purified hormones used for HPLC reference standards or for testing as potential cross reactants (Sigma Aldrich, St. Louis, MO, or Fisher Scientific, Hampton, NH) include: EtOH (Ethanol 200 proof, #459828), MeOH (Methanol, #A452-1), acetonitrile with 0.1% TFA [v/v, Trifluoroacetic Acid (v/v), Optima™ #6000063], T2 (3,3′-Diiodo-L-thyronine, #719536), T3 (3,3′,5-Triiodo-L-thyronine, #T2877), T4 (L-Thyroxine, #T2376), Iodide (certified reference material #41271, 1,000 mg/L); and the following certified reference materials for thyroid standards [Cerilliant® 100 μg/mLin Methanol: T-073 T4, T-074 T3, and T-075 reverse T3 (rT3)].

#### 2.3.2 Thyroid tissue extraction

Historically, thyroid hormone testing in SR serum samples had produced mostly low or near zero values. As a biological validation, thyroid tissue was retrieved from a wild-caught (bycatch) SR (Hendon) and stored frozen at -80C. Thyroid tissue was thawed, homogenized and 0.25 g was extracted with ice cold MeOH, centrifuged and the supernatant was passed through a syringe filter before HPLC fractionation (25–27).

#### 2.3.3 High performance liquid chromatography

Reverse-phase HPLC was used to separate thyroid hormones of interest, fractioning samples for subsequent RIA evaluation of immunoreactivity within each fraction. HPLC methods followed Sweeting and Eales (28), also see (29, 30), and utilized a Dionex Ultimate 3,000 set up (31) with the following changes for thyroid analyses. Solution A contained water with 0.1% TFA, and solution B contained acetonitrile with 0.1% TFA (v/v, #6000063). Following column equilibration in Solution A, 100 μL of the sample was injected into the column with a flow rate of 0.75 mL min-1. Hormones were collected at a linear gradient of 34 to 45% of solution B over 30 min followed by a pulse of 5 min ACN to clear residual lower polarity steroids. Elution peaks were determined following injection of concentrated Iodide, T2, and analytical reference standards (rT3, T3, T4, 100 μg/mL), monitored at 190, 220, 240 and 254 nm. Singly injected standards often eluted with earlier retention times (RT) than the pooled panel of standards, so the range or medians of RT are reported where appropriate. Fractions (fr) were collected every 60 s from 1 to 29 min into tubes, evaporated, and reconstituted in 1 mL of heat-inactivated, charcoal stripped FBS (fetal bovine serum albumin) as diluent for the RIA. Fractions were shipped frozen overnight along with the FBS diluent, Iodide, rT3 and T2 standards to MSU VDL (Michigan State University, Veterinary Diagnostic Laboratory) for testing and cross-reactivity assessment in the thyroid RIA panel via the standard protocols, using 100 μL for TT3, 200 μL for TT4, and 50 μL for FT4.

#### 2.3.4 Blood samples

Thyroid Radioimmunoassay (RIAs): Optimizations for MSU VDL RIA thyroid panel (#20015).

#### 2.3.5 Preliminary data

Upon initial analysis, only a small percentage of the lagoon or wild animal samples were within the readable range of the standard curve when analyzed using standard protocols and sample volumes. As part of the MSU VDL standard protocol, low concentration samples are provided values following extrapolation from the lowest standard to zero. To improve detection accuracy, samples were rerun with increased serum volumes (where possible), using additional lower concentration standards down to the reported assay sensitivity limit to allow for interpolation of lower sample analyte concentrations (vs extrapolation). Where appropriate, the RIA analysis program was edited to increase decimal units to improve precision (reduce rounding). Banked SR blood samples from (previously determined) high TT3, TT4 sample values were pooled for tests of parallelism of resultant values when testing multiple sample volumes in the RIAs (TT3, TT4, see below for details). Curve fitting was used to interpolate thyroid values using the extended standards with and without the extended volumes to compare against extrapolated values.

#### 2.3.6 Total triiodothyronine (TT3) by RIA

The TT3 assay was run using an in-house RIA. TT3 standards ranged from 0.4 nmol/L to 4.7 nmol/L. TT3 RIA sensitivity was reported as 0.76 μg/dL (or 0.049 nmol/L). Standard sample test volume used in the assay was 100 μL, run in duplicate. For serum samples with resultant values below the lowest provided standard (0.4 nmol/L) a value was estimated through standard extrapolation between 0.4 nmol/L and zero. For assay optimization of TT3 for use in elasmobranchs we tested and compared increased sample volume (100 and 200 μL), and included an additional three lower standards (0.20, 0.10, and 0.05 nmol/L) to compare values obtained by extrapolation vs. interpolation using the expanded standard curve.

#### 2.3.7 Total thyroxine (TT4) by RIA

Total T4 (TT4) was measured using a commercially available radioimmunoassay kit (MP Biomedicals Diagnostics Division). Total T4 standards ranged 25.7 nmol/L to 257.0 nmol/L. Sensitivity was reported as 0.76 ug/dL (or 0.059 nmol/L TT4). Sample volume was 25 μL, run in duplicate. For serum samples with resultant values below the lowest provided standard (25.7 nmol/L) a value was estimated through standard extrapolation between 25.7 nmol/L and zero. For assay optimization, an increased sample volume (100 and 200 μL) was used and 3 additional lower standards (12.9, 6.45, and 3.23 nmol/L) were added to compare extrapolated vs. interpolated results.

#### 2.3.8 Free thyroxine (FT4) by equilibrium dialysis followed by T4 RIA

Free T4 (FT4) was assessed by equilibrium dialysis and was measured by a commercially available radioimmunoassay manufactured by Antech Diagnostics. T4 standards ranged 3.6 to 126 pmol/L. Sensitivity was reported as 0.045 ng/dL (0.58 pmol/L). Sample test volumes were 50 μL, run in duplicate. For serum samples with resultant values below the lowest provided standard (3.6 pmol/L), the value was estimated through standard extrapolation between 25.7 nmol/L and zero. For assay optimization, the sample volume could not be increased due to equilibrium dialysis methods; however, the software was altered to provide two decimals, reducing rounding. Three additional lower standards (1.8, 0.9, and 0.45 pmol/L) were included to compare extrapolated vs. interpolated results.

#### 2.3.9 Thyroxine (T4) for TT4 chemiluminescent immunoassay

Total thyroxine (TT4) was measured with a commercially available solid-phase competitive chemiluminescent enzyme immunoassay (Total T4 Chemiluminescent Immunoassay, Immulite® 2000XPi, Siemens Healthcare, Glyn Rhonwy, Llanberis, United Kingdom). The TT4 CLIA assay protocol was run via an automated instrument. The instrument picks up the volume it requires for the test (100 μL), and therefore sample volume was unable to be modified for testing. A subset of 54 samples were run on the TT4 CLIA to be compared to the optimized TT4 RIA.

### 2.4 Iodine testing

Serum for total (the sum of inorganic and organic/protein-bound) and inorganic iodine was submitted to the MSU VDL for analysis as per Wahlen et al. (32).

### 2.5 Elemental analysis including cobalt

Serum was submitted to the MSU VDL for cobalt analysis as part of a trace mineral panel, using inductively coupled plasma-mass spectrometry (ICP-MS). Standards were from Inorganic Ventures. In-house serum pools were used as controls.

### 2.6 Ultrasound evaluation

Thyroid ultrasounds were reviewed by one author (Mylniczenko). The purpose of using this tool in this study was to identify animals that had grossly different thyroid appearance from known normal animals, suggesting disease as identified by experience from the authors and in comparison to other species. Images were identified retrospectively from exams which were conducted by multiple individuals; they were not always done with uniform technique and quality, thus acquisition of consistent measurement parameters was not possible. There are limited references on ultrasound evaluation of thyroid glands in elasmobranchs (33). The shape and size of the thyroid varies among species and can be difficult to identify (34). A normal thyroid has a symmetrical, homogenous appearance (35, 36) and in elasmobranchs they are smaller or equal in size to adjacent coracohyal and coracomandibular musculature (34, 37). Any variation from this uniformity was documented.

For a basic or ‘high level’ assessment of thyroid disease, the following criteria were used:

(1) the thyroid was larger than the adjacent musculature.
(2) multiple cysts were present.
(3) there were scattered hyperechoic foci present.

Cysts and foci were not quantified, they were subjectively evaluated and listed as mild, moderate, and severe.

### 2.7 Statistical analyses

All analyses were performed using R statistical Software v4.3.2 (38) or SigmaPlot (v.14.0), or MedCalc software Ltd. v.23.0.9. All statistical tests were evaluated at an 𝛼< 0.05.

Quantitative measures (thyroid, cobalt, and iodine) were tested for normality using the Shapiro–Wilk normality test and visual inspection of the data normality using Q-Q plots (quantile-quantile plots) in the R ‘stats’ package (38). Since most of the data were left skewed and failed tests for normality even after standard transformations, comparisons between habitats and study cohorts were evaluated using non-parametric methods and corrections were applied for multiple *post-hoc* tests as appropriate. Log-transformation was employed on iodine measures during linear and ordinal regression model testing and evaluations. Single pairwise comparisons of serum thyroid, cobalt, or iodine measures evaluating differences between species and habitats used Mann–Whitney Rank Sum Tests. Study cohorts and habitat results were summarized as median (χ̃) and interquartile range [IQR; 25th and 75th percentile (%)], where appropriate.

#### 2.7.1 Wild and lagoon-housed normal populations

The thyroid and iodine values from “normal” thyroid (as determined by ultrasound) wild and lagoon-housed SRs were compared using Kruskall-Wallis one way ANOVA on ranks, and Dunn’s Method for all (*post hoc*) pairwise comparisons. Comparisons between species, habitat, and supplement type were plotted using the web app “PlotsOfData.” Data are displayed with underlying distributions, ordered by medians with mean and 95% confidence intervals by bootstrapping [see (39)].

#### 2.7.2 Correlation analyses of serum thyroid, cobalt and iodine measures (cohort 1 and 2)

All pairwise correlations from all thyroid scores between quantitative sample hormone data (iodine (inorganic), iodine (total), cobalt, TT3, TT4, FT4) were evaluated using Spearman’s rho statistic (⍴), rank-based correlation tests of all pairwise complete observations using the R packages ‘corrplot’ and ‘Hmisc’ to compute the *p* values of the correlations (38, 40–42). Variables for the correlogram were arranged in the angular order of the eigenvectors (AOE) which are ordered to match their corresponding eigenvalues, sorted in ascending order by magnitude (42). Significant correlations are reported as ⍴(df) = [⍴ value], *p* = [*p*-value], where df = N-2.

#### 2.7.3 Regression analysis of thyroid hormone, iodine, and cobalt measures and thyroid disease states (cohort 2)

Principal component analysis and factor analysis were initially used to explore relationships between thyroid iodine and cobalt measures within cohorts to identify important factors (supplementation and thyroid disease severity) and drive model selection (see Supplementary materials). For model selection, serum thyroid hormone, iodine, and cobalt data from aquarium-managed animals were first assessed for violations of model assumptions, multicollinearity, and VIF (variance inflation factor). VIF was also used to aid in model selection, and trim predictor variables. The associations of thyroid hormone, iodine, and cobalt measures with thyroid disease were first investigated using simple linear regression models, followed by ordinal regression (see Supplementary Table S3 for additional details). For this analysis, ultrasound evaluations of the thyroid at the time of blood sample collection were transformed into an ordered-factor (normal, normal/large =1, goiter =2, mild =3, moderate =4, moderate–severe =5) for use in multivariable proportional odds logistic regression modeling. Ordinal regression model output provided coefficients given in units of ordered logits (ordered log odds). For a simpler interpretation, we converted coefficient values into an odds ratio (OR; the inverse log of the estimated coefficients). Significant coefficients in the model are reported as OR and 95% confidence interval (CI).

In a second model, we were interested in the predictive effects of total iodine measures. For this analysis, total iodine data was transformed into a bivariate categorical variable (< or > 400 ng/mL) alongside thyroid hormone, inorganic iodine, and cobalt (reporting probability estimates of each thyroid disease level). Predictor effect plots of significant factors in the model (coefficients) were built using the ‘effects’ package in R (43, 44).

## 3 Results

### 3.1 Thyroid assay optimization and validation

#### 3.1.1 TT3

Detection and accuracy in measurement of thyroid hormones was improved by increased sample volume and use of additional lower standards. Visual inspection confirmed parallelism of calculated values with normal and extended volumes (100 vs. 200 μL, sample TT3 values >1.0 nmol/L) and from normal standards and extended standards. In a subset of test samples (*N* = 16 SR from the wild cohort), only 33% of the samples (5 of 15) fell within the range of the standard curve using the standard protocol (100 μL sample volume), whereas, 81% (13 of 16 samples) were within the readable range of the standard curve with increased volume (200 μL) and extended standards. Sample TT3 values using 100 μL volume obtained by extrapolation (from the lowest standard to zero) were strongly correlated with result values obtained via interpolation using 200 μL sample volume and additional lower standards (r^2^ = 0.985).

#### 3.1.2 TT4

TT4 detection was improved by increasing sample test volume from the standard 25 μL to 200 μL. No samples (0 of 16) were detected within the readable range of the standard curve using 25 μL sample volume (normal TT4 standards ranged 25.7 nmol/L to 257.0 nmol/L). Increasing sample volume to 200 μL (8x increase), and adding additional lower standards improved detection to 68.8% (11 of 16). Comparison of extrapolated vs. interpolated values for 200 μL sample volume showed a strong positive correlation (r^2^ = 0.99), indicating that the extrapolation was sufficient, but the extra sample volume was a critical improvement.

#### 3.1.3 FT4

Serum volumes could not be increased at the dialysis step, so FT4 optimization was only evaluated for improved detection based on extended standards. None of the samples (0 of 16) fell within the readable range of the standard curve using the 50 μL sample volume and regular standards. Adding additional lower standards increased sample detection on the curve to 25% (4 of 16). Ten samples had reported values of 0 pmol/L. However, values determined by extrapolation were strongly correlated with interpolated values (r^2^ = 0.98).

Overall, strong, positive correlations for extrapolated vs. interpolated values in all three thyroid RIAs indicated that including extra standards could improve detection but were not necessary for improved accuracy for normal elasmobranch serum sample values at or near the sensitivity of these assays. However, higher sample volumes (TT3 and TT4) significantly improved detection of low thyroid concentrations.

#### 3.1.4 CLIA vs. RIA (TT4)

Due to discontinuation of the RIA TT4 kit near the completion of this study, a small subset of samples (*N* = 54) with a range of serum TT4 RIA values (200 μL test volume), across multiple species, were tested in the new TT4 CLIA platform. A 100 μL test volume was used, which could not be increased due to limitations in assay methodology. Most of the CLIA values (44 of 54) were reported with zero values (vs. only 8 from RIA) resulting in both measures to have a non-normal distribution with positive skew (Shapiro–Wilk RIA TT4, W = 0.8016, *p* < 0.0001; CLIA TT4 W = 0.2842, *p* < 0.0001). The non-zero values ranged from 0.014 to 6.83 nmol/L for RIA vs. 0.193 to 15.2 nmol/L for the CLIA, However, serum TT4 CLIA values did not correlate with the TT4 from RIA (adjusted r^2^ = −0.0148). Data were next checked for assumptions of normality of differences for Bland–Altman analysis (MedCalc). The difference in TT4 measurements (RIA-CLIA) revealed an overall positive bias with the optimized RIA TT4 method reporting higher values than the CLIA (RIA-CLIA difference mean ± SD was 0.95 ± 2.93 nmol/L). However, since RIA-CLIA differences were not normally distributed (Shapiro–Wilk W = 0.7801, *p* < 0.001), further evaluation using Bland–Altman plots could not be conducted. Removal of the zero-reported values to re-analyze the data was not done since the size of the remaining dataset (*N* = 10) would reduce statistical power and limit interpretation. Because of the poor correlation and repeatability between methods, no further testing or analysis of CLIA TT4 with respect to thyroid disease or the other measures were performed.

#### 3.1.5 HPLC

**Figure 1:**
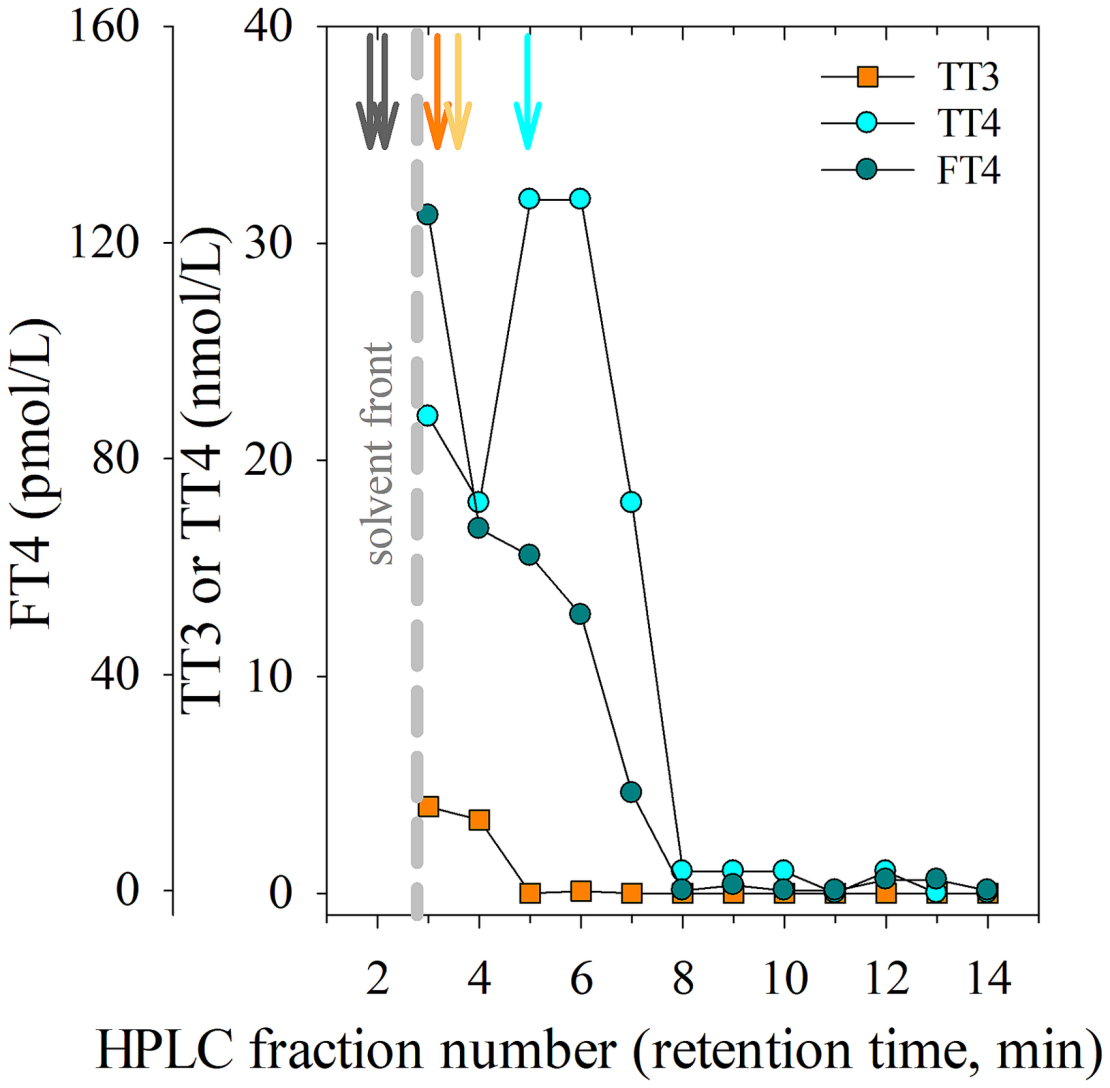
High performance liquid chromatography (HPLC) chromatogram including fraction (fr) number and retention time (RT) peaks from injection of methanolic iodide and thyroid standards (denoted by vertical arrows) or immunoreactivity results from fractions collected following injection of a methanolic extract of thyroid tissue (collected from *H. americanus*) run in commercially available RIAs (radioimmunoassay; TT3; total triiodothyronine, nmol/L, orange square); TT4 (total thyroxine, nmol/L, cyan circle; FT4; free thyroxine, pmol/L, dark teal circle). Reference standard RTs are depicted as vertical arrows shown in order from highest to lowest polarity: Iodide and T2 (dark brown), T3 (orange), rT3 (light orange), T4 (cyan). The solvent front is denoted by a vertical gray dashed line.

Results from a HPLC chromatogram, including peak RT of the Cerilluant thyroid standards and iodide, and RIA immunoreactivity profile of the thyroid methanolic extract (see Figure 1) was used to characterize immuno-detectable TT3, TT4 and FT4 (see Supplementary Figure S1 for additional details). Two standards (I2 and T2) partially co-eluted with the wavefront and had RTs as follows: iodide (I2) in a wide band at 1–1.83 RT, T2 (2.073–2.683 RT), whereas T3 and T4 separation was more distinct [T3 2.1 RT (single), 3.78–4.033 RT (panel)], rT3 [3.9–4.1 (single), 4.28–4.55 RT (panel)], and T4 [4.18–4.44 (single), 5.6–6.09 RT (panel)]. Thyroid tissue extract produced a wide band of immunoreactivity starting at the wave front in fr1-2 and continuing through fr 3–4. The highest relative percent immunoreactivity in the TT3 assay was found in a wide band starting at the wave front in fr 1–2 and continued, in part, into fr 3. An unexpected and unidentified immunoreactivity peak was observed in fr 2–3 in TT4 and FT4 RIA. Cardiac blood collected from the wild animal at the time of thyroid tissue recovery had low concentrations of TT3, TT4 and FT4 (0.00 nmol/L, 1.13 nmol/L, and 0.00 pmol/L, respectively).

#### 3.1.6 Assessment of thyroid ‘normals’ using the optimized thyroid RIAs, cobalt, and iodine

**Figure 2:**
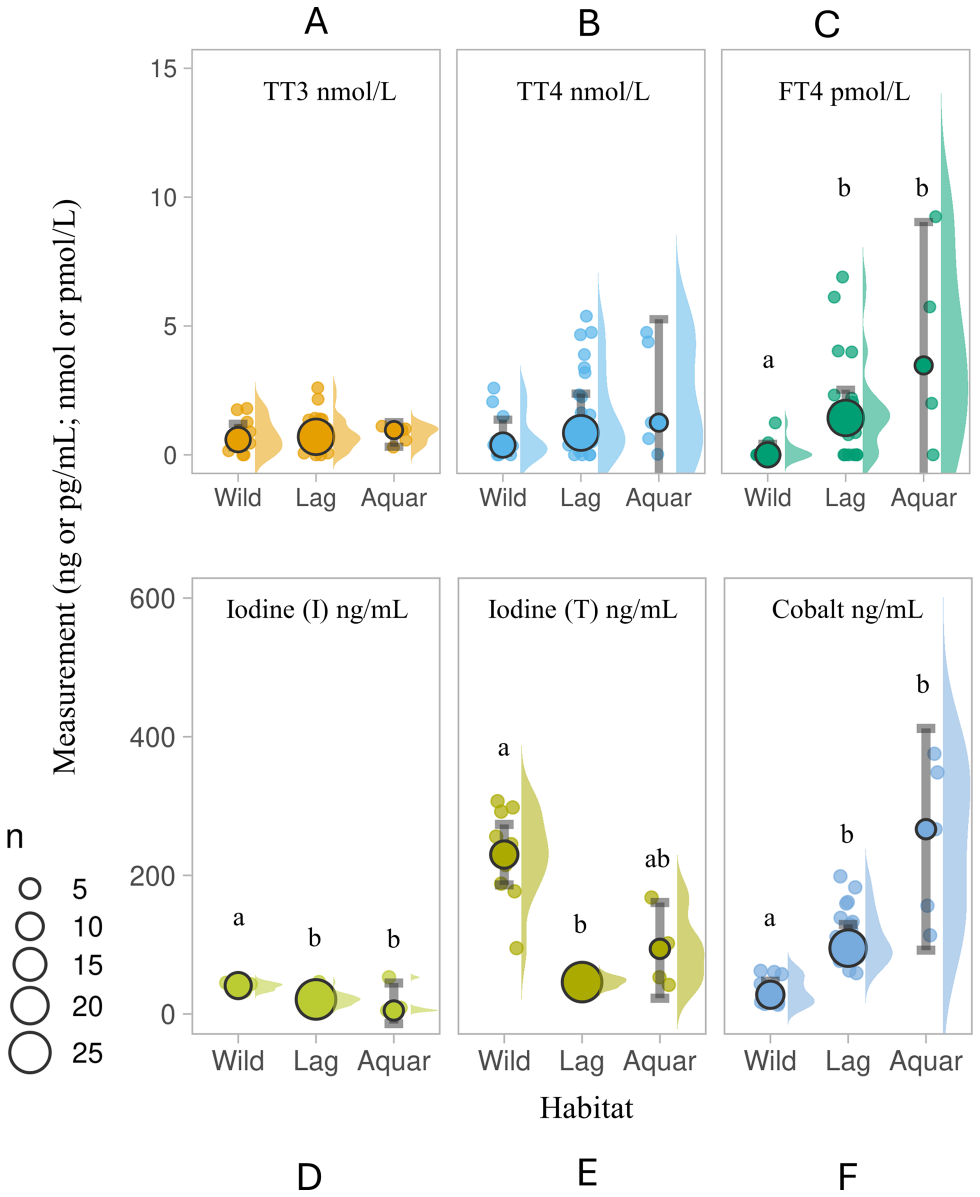
Serum **(A)** total triiodothyronine (TT3 nmol/L), **(B)** total thyroxine (TT4, nmol/L), **(C)** free thyroxine (FT4, pmol/L), **(D)** inorganic iodine (I; ng/ml), **(E)** total iodine (T; ng/ml), and **(F)** cobalt (ng/ml) in three populations of wild and housed southern stingrays *Hypanus americanus* with normal presentation of thyroids by ultrasound evaluation (Study 1). Data are displayed as individual sample dot-plots (jittered) with vertical cow-plots to visualize the data distribution for each measure by habitat [(Wild, lagoon (Lag), aquarium (Aquar)]. Medians for each habitat are indicated by the larger outlined circles, with the relative size of the circle indicating the N of that habitat (see key). Error bars represent the 95% confidence interval for the habitat. Letters inside the panels represent significant differences between the habitat (α < 0.05). Wild animals (*N* = 11) were unsupplemented. Lagoon animals (*N* = 22) received Aquatic Gel, and 1 of 5 Aquarium animals received supplement “C” (Table 1).

Wild, lagoon- and aquarium-housed thyroid-normal SR did not differ in TT3 (TT3 χ̃=0.59, 0.70 and 0.46 nmol/L, respectively) or TT4 values (TT4 χ̃=0.38, 0.85 and 0.47 nmol/L, respectively), however FT4 values were higher in the lagoon (FT4 Lag χ̃=1.43 pmol/L, IQR = 0.39, 2.12) and aquarium-housed (FT4 Aquar χ̃=0.00 pmol/L, IQR = 0.00, 1.90) SR compared to the wild SR (FT4 Wild χ̃=0.0 pmol/L, IQR = 0.0, 0.0; H (2)=11.961; *p* = 0.003; see Figures 2A–C).

SR serum iodine (total, inorganic) and cobalt measures showed more variability. Wild SR had higher serum inorganic iodine relative to lagoon- and aquarium-housed SR (H (2)=28.054, *p* < 0.001), and higher total iodine (I (T) Wild χ̃=230.0 ng/mL, IQR = 188.0, 292.0, all values <400 ng/mL) than lagoon-housed SR (H (2)=29.765, *p* < 0.001). Lagoon- and aquarium-housed SR had elevated serum cobalt measures compared to wild SR (H (2)=8.342, *p* = 0.015). Cobalt values in the wild SR group (*N* = 11) ranged from 13.41 to 62.20 ng/mL, whereas lagoon- and aquarium-housed SR ranged from 58.98 to 198.60 ng/mL (*N* = 25) and 113.50 to 375.40 ng/mL (*N* = 5), respectively (see Figures 2D–F). Four of the five aquarium-housed SR (all females) were 80 days post-removal of supplement “C” (3.8 ppm DM cobalt).

### 3.2 Iodine and cobalt measures across all cohorts and thyroid disease states

**Figure 3:**
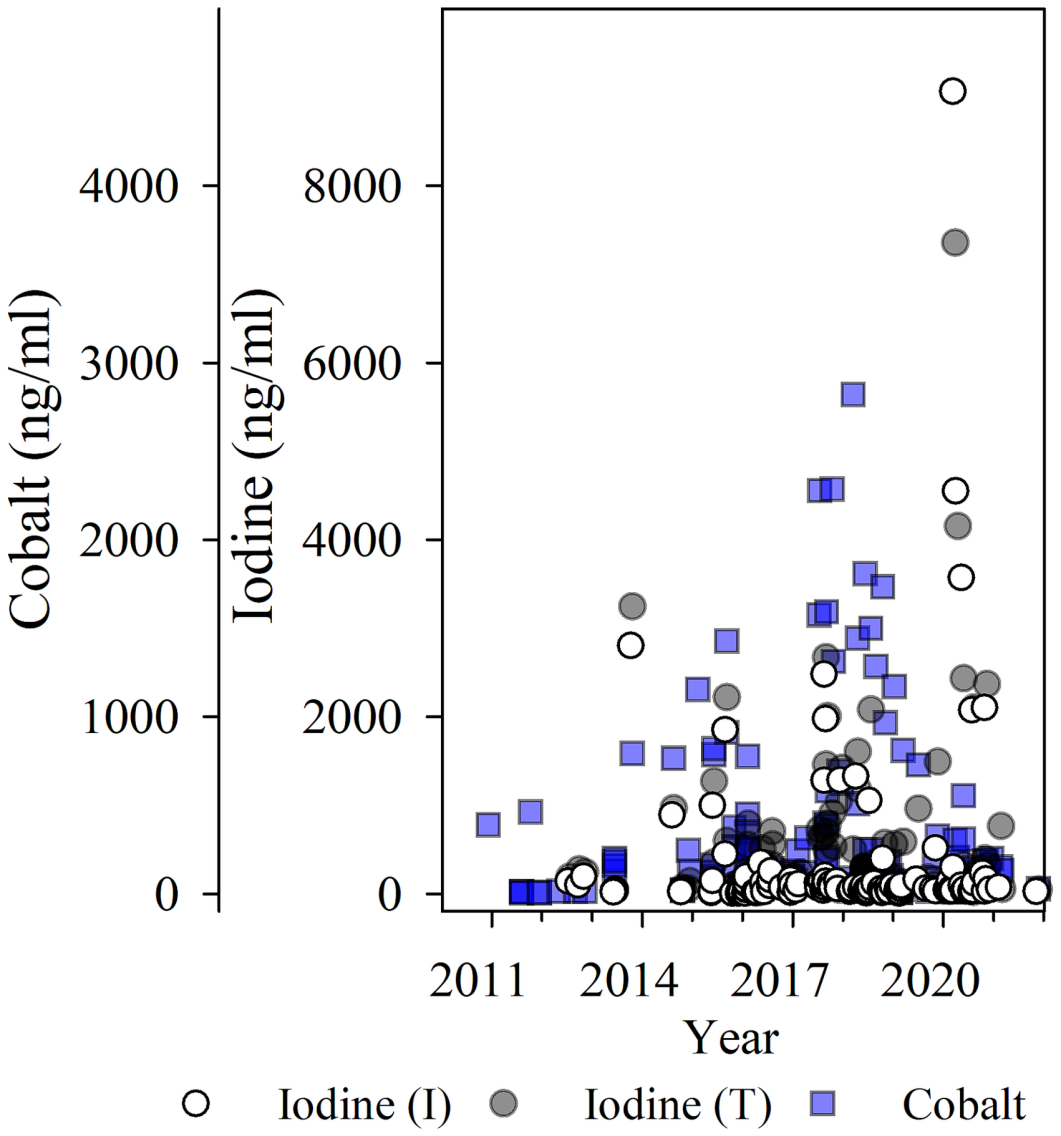
Serum iodine [inorganic (I) or total (T) ng/ml] and cobalt (ng/ml) values plotted for study years 2010 to 2022 from both Study cohorts provided the supplementation described in Table 1. Two samples with extremely high iodine values >10,000 ng/mL are not shown (*N* = 2; inorganic iodine: 16,642 and 606,740 ng/mL; total iodine: 17,257 and 493,529 ng/mL). This graph shows that use of supplementation appears to accumulate iodine and cobalt across time.

Iodine values measured as high as 606,740 ng/mL [I (I); N = 151 χ̃=48.0 ng/mL, IQR = 25.00, 130.00] for inorganic iodine, and 493,529 ng/mL [I (T); N = 157, χ̃=150.0 ng/mL, IQR = 53.00, 495.00] for total iodine in WSBS with moderate to severe thyroid disease. Cobalt ranged from 4.24 to 2,818.91 ng/mL (*N* = 146, χ̃=112.33, IQR = 27.78, 316.17; see Figure 3). The highest iodine and cobalt values (>1,000 ng/mL) were observed in aquarium-housed animals in 2015 through 2020 (*N* = 15), and primarily in WSBS species (12 of 15 samples, *N* = 2 individuals, samples collected from 2013 to 2020). These high values were obtained when the WSBS were offered (and consumed) supplement “C” and “D” with approximately 100,000 ppm DM iodine (see Table 1). Highest cobalt values were observed in 2017 and 2018 when animals were offered supplement “D” (5M1Y; 5MD8) approximately 2 years after iodine content had been reduced by over 33% from 202,603 ppm DM down to 133,658 ppm DM, and more than 5 years after cobalt had been reduced from 151.4 ppm DM (supplement “A”) down to 4.2 ppm DM (supplement “B”) and 3.8 ppm DM (supplement “C”; see Table 1). Only 29 of 53 samples with values <300 ng/mL cobalt were evaluated as thyroid-normal. The remaining samples included 5/53 goiter; 9/53 Normal (LG); 5/53 mild; 5/53 moderate; and 8/53 moderate–severe. There was a mix of thyroid disease scores in the higher cobalt measures (>300 ng/mL), including 7/27 evaluated as normal, 6/27 Normal (LG), 0/27 goiter, 9/27 mild, 1/27 moderate, and 4/27 moderate–severe thyroid disease. The highest cobalt values observed (*N* = 13 > 700 ng/mL) primarily came from HCR, PR, and WSBS species. The lowest iodine and cobalt values were observed in wild and lagoon-housed SR (see Figures 2D–F), where all measured total iodine values were < 400 ng/mL, inorganic iodine values were < 50 ng/mL, and cobalt values were < 200 ng/mL. There was one exception in an aquarium-managed HCR with undetermined thyroid disease status, measuring a low of 14 ng/mL for both inorganic and total iodine.

### 3.3 Investigation of overall relationships between thyroids, iodines, and cobalt measures

**Figure 4:**
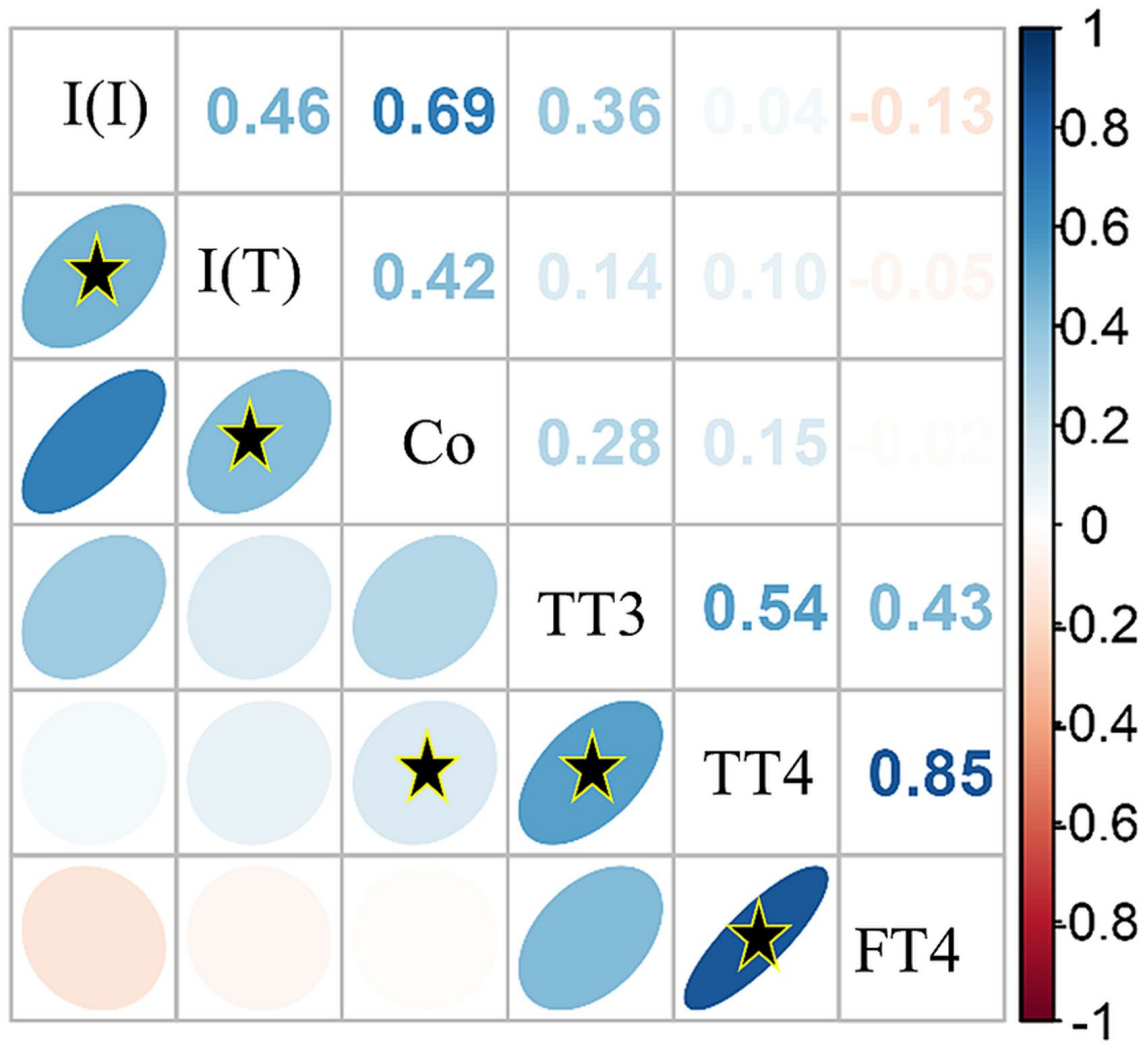
Correlation matrix of serum inorganic iodine [I (I)], total iodine [I (T)], cobalt (Co) and thyroid hormone values [total triiodothyronine (TT3); total thyroxine (TT4); free thyroxine (FT4)] using Spearman’s correlation method with all pairwise complete observations. Color intensity is proportional to the correlation coefficients. Direction of the ellipses indicates positive or negative relationships. On the right side of the correlogram, the legend color relates to the Spearman correlation coefficient values, with blue indicating positive and red indicating negative correlations. The correlation matrix was reordered according to the correlation coefficient using the angular order of the eigenvectors (AOE) method. Black stars (*) within ellipses indicate significant correlations with α < 0.01.

Spearman’s rank correlations were computed to assess all available pairwise relationships between quantitative measures. Overall, there were strong, significant positive relationships between inorganic iodine and total iodine (⍴(149) = 0.85, *p* < 0.0001), total iodine and cobalt (⍴(131) = 0.54, *p* < 0.0001), TT4 and FT4 (⍴(112) = 0.69, *p* < 0.0001). Significant but weaker relationships were also observed between TT3 and TT4 (⍴(111) = 0.42, *p* < 0.0001), and TT3 and cobalt (⍴(45)=0.14, *p* < 0.0001; see Figure 4). All other correlations were of lower strengths of effect, but it was observed that in this correlation matrix, inorganic iodine had a weak, negative correlation with two of the thyroid measures (TT3 ⍴(46)= − 0.05, and FT4 ⍴(47)= − 0.14, see Figure 4, light red negative ellipses).

### 3.4 Ultrasound evaluation

#### 3.4.1 Ultrasound evaluation and thyroid disease evaluation score

**Figure 5:**
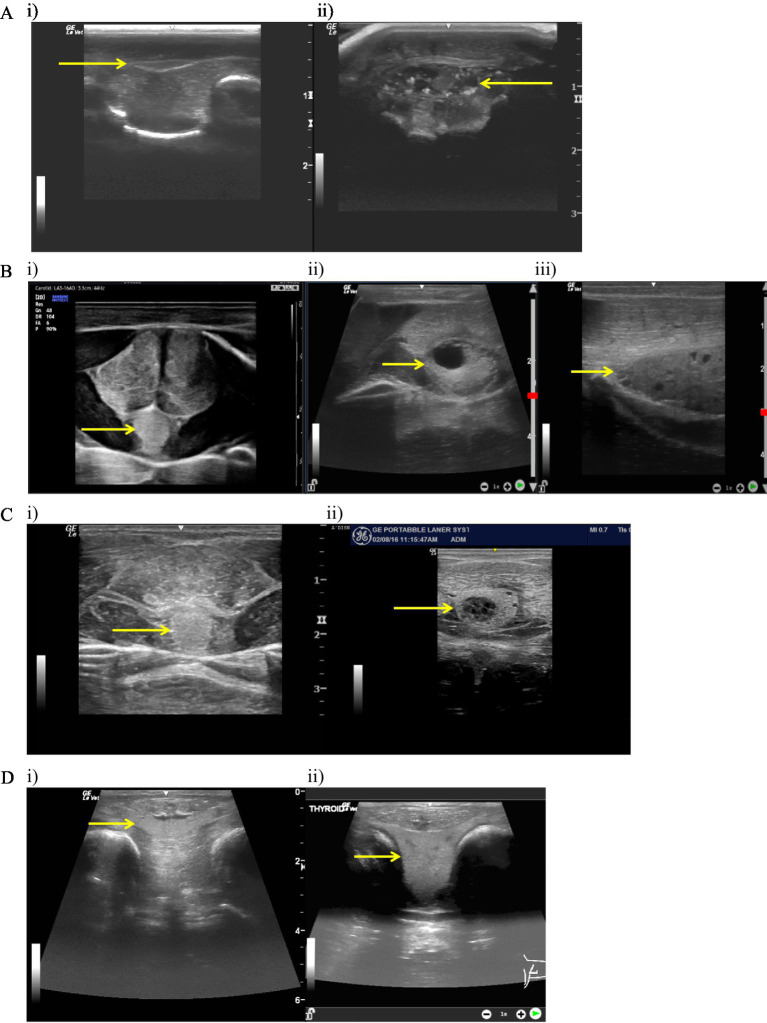
Ultrasound evaluations. (**A**: i) Normal bamboo shark (*Chiloscyllium punctatum*) thyroid ultrasound in transverse; arrow scaled to 1 cm. (ii) Abnormal bamboo shark (*Chiloscyllium plagiosum*) thyroid ultrasound in transverse; arrow scaled to 1 cm. Complex multinodular cystic appearance with hyperechoic foci. (**B**: i) Normal honeycomb stingray (*Himantura uarnak*) thyroid ultrasound in transverse; arrow scaled to 1 cm. (ii) Abnormal honeycomb stingray (*H. uarnak*) thyroid ultrasound in transverse; arrow scaled to 1 cm. Note the large cyst present and the overall enlarged size of the thyroid. (iii) Abnormal honeycomb stingray (*H. uarnak*) thyroid ultrasound in sagittal; arrow scaled to 1 cm. Note multiple small cystic structures present throughout the thyroid and larger size compared to adjoining musculature. (**C**: i) Normal southern stingray (*Hypanus americanus*) thyroid ultrasound in transverse; arrow scaled to 1 cm. (ii) Abnormal southern stingray (*H. americanus*) thyroid ultrasound in transverse; arrow scaled to 1 cm. Note the complex cystic nodule with smaller cysts in the parenchyma and the size of the thyroid compared to the ventral musculature. (**D**: i) Normal ocellated eagle ray (*Aetobatus ocellatus*) thyroid ultrasound in transverse; arrow scaled to 1 cm. (ii) Abnormal ocellated eagle ray (*Aetobatus ocellatus*) thyroid ultrasound in transverse; arrow scaled to 1 cm. Note the hyperplasia (goiter).

The most common findings (see Figure 5) were cysts of varying sizes and hyperechoic pinpoint foci, likely representing mineralization (48). Nodular disease with hypoechoic cystic (spongiform/honeycomb) appearance was labeled as moderate disease based on size of the gland and the cyst.

Extent of thyroid disease was broad and evaluation was intended only to assess if there was any correlation with blood data and individuals with a history of reproductive disease (*N* = 4 SR; *N* = 2 WSBS; *N* = 4 HCR). Histopathologic confirmation of thyroid disease was not typically possible in this dataset as biopsies were rarely taken and postmortem thyroid tissue was not always included in the sampling set. A summary of thyroid evaluation scores at the time of blood sampling are provided in Supplementary Table S2.

**Figure 6:**
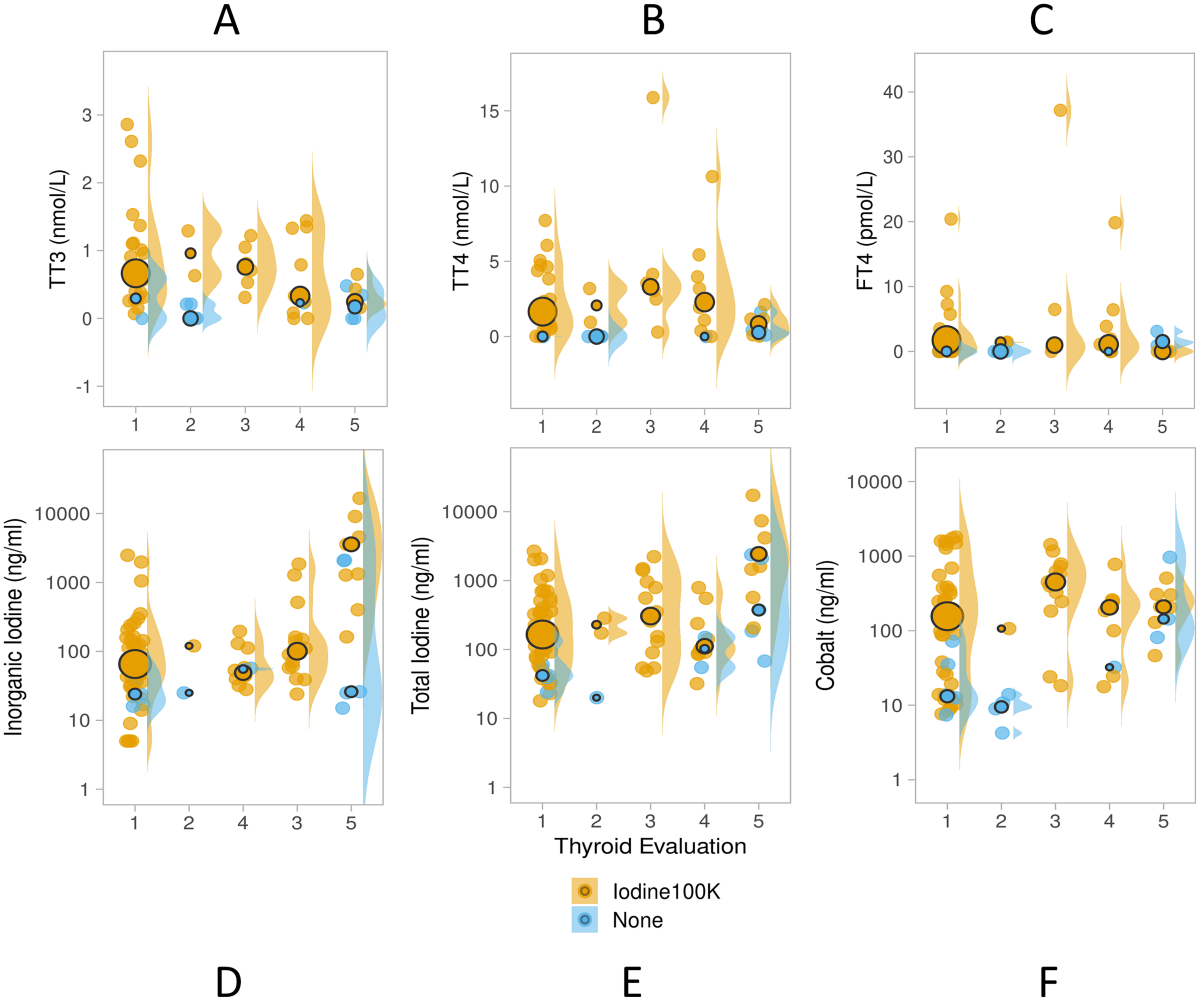
Study 2 serum TT3 (nmol/L), TT4 (nmol/L), FT4 (pmol/L; panels **A–C**), inorganic iodine (ng/ml), total iodine (ng/ml) and cobalt (ng/ml; panels **D–F**) values in unsupplemented (blue, *N* = 20) and micronutrient supplemented elasmobranchs (orange, *N* = 82) with total iodine dose ~100,000 ppm DM (Iodine 100 K), and cobalt dose 3.8 or 1.4 ppm DM (Supplements “C” and “D,” Table 1.) Serum inorganic, total iodine and cobalt shown here with y-axis in log-scale. Thyroid evaluations obtained by ultrasound during the time of the blood sample collection provided a range of thyroid disease from 1 = normal, or normal/large, 2 = goiter, 3 = mild, 4 = moderate, and 5 = moderate/severe. Data are displayed as individual sample dot-plots (jittered) with vertical cow-plots to visualize the data distribution for each measure. Medians for each group are indicated by the larger outlined circles, with the relative size of the circle indicating the N of that group.

Samples collected from animals in natural sea water habitats (wild or lagoon-housed) were categorized as normal based on relative size and homogenous symmetrical appearance, whereas aquarium-housed animals provided a range of thyroid disease scores (see Supplementary Table S2). Thyroid, cobalt, and iodine measures differed across thyroid disease evaluation levels, with moderate and moderate/severe thyroid evaluations showing the highest total and inorganic iodine values, but lowest TT3 (see Table 2). Distributions of measured values of TT3, TT4, and FT4 shifted to higher ranges (increased variance; larger interquartile ranges) in supplemented animals (see Figures 6A–C). The pattern was consistent through disease states, with the notable exception of animals in the highest thyroid disease category (thyroid evaluation =5, moderate/severe), which did not show the same pattern of elevated TT3, TT4 or FT4 (Figures 6A–C).

**Table 2:** Comparisons of SR thyroid, iodine, and cobalt medians and interquartile range (IQR) across thyroid disease evaluation scores (number) for each study elasmobranch cohort (natural sea water or aquarium habitat).

Habitat	Thyroid ultrasound evaluation							
	Natural sea water		Aquarium					
Measurement^a^ median (IQR^b^)	Normal (1) (Wild)	Normal (1) (Lagoon)	Normal (1)	Normal/ Large (1)	Goiter (2)	Mild (3)	Moderate (4)	Moderate/ Severe (5)
TT3 (nmol/L)	0.59 (0.16, 1.27)	0.70 (0.36, 1.18)	0.46 (0.25, 0.95)	0.75 (0.30, 2.34)	0.21 (0.00, 0.57)	0.76 (0.48, 1.09)	0.29 (0.06, 1.34)	0.25 (0.12, 0.44)
TT4 (nmol/L)	0.38 (0.00, 1.49)	0.85 (0.41, 2.98)	0.47 (0.00, 2.23)	1.66 (0.24, 4.43)	0.00 (0.00, 0.94)	3.31 (1.940, 7.07)	2.12 (0.29, 4.34)	0.52 (0.11, 1.62)
FT4 (pmol/L)	0.00 (0.00, 0.00)	1.43 (0.39, 2.12)	0.00 (0.00, 1.90)	2.09 (0.23, 2.69)	0.00 (0.00, 1.03)	0.97 (0.47, 14.14)	0.96 (0.00, 4.51)	0.00 (0.00, 1.62)
Inorganic iodine (ng/ml)	41 (35, 44)	21 (14, 27)	54 (33, 137)	83 (17, 164)	96 (25, 120)	90 (40, 161)	54 (41, 98)	1,306 (38, 4,307)
Total iodine (ng/ml)	230 (188, 292)	46 (40, 56)	99 (55, 267)	323 (42, 698)	224 (58, 283)	258 (54, 960)	112 (86, 216)	1,532 (247, 3,723)
Cobalt (ng/ml)	27.78 (16.23, 57.44)	95.03 (80.87, 135.75)	94.64 (12.10, 247.26)	114.10 (25.90, 1284.06)	10.11 (7.78, 37.07)	423.68 (144.31,740.92)	195.64 (30.36, 254.75)	177.27 (93.01, 306.18)

a Total triiodothyronine (TT3; nmol/L); Total tetraiodothyronine (TT4; nmol/L); Free thyroxine (FT4; pmol/L). Sample Ns are provided in Supplementary Table S2.

b Interquartile range (IQR) reported here as: (25th, 75th percentile).

Several of the aquarium-housed individual animals (*N* = 38) in this study were monitored over several years. There was a progression of thyroid disease and an opportunity to see if it resolved. Four SER presenting with hyperplastic goiters resolved with management. One animal was provided supplementation (supplement “A,” Tables 1, 3) were given oral lugol’s iodine until the goiter resolved and were then put on the supplement. In all other cases, during the course of the study, progression from a normal thyroid evaluation to mild, moderate or moderate–severe was unidirectional.

**Table 3:** (A) Ultrasound evaluation of elasmobranch thyroid gland architecture including presence or absence of cysts and hyperechogenic foci, used to provide an overall measure of overall thyroid normality score during the time of blood sampling in all habitats.

	Total iodine group (ng/ml)		Overall	*p*-value^a^^,^^e^
	< 400	>400		
(A) Ultrasound evaluation N(%)				
Cysts				<0.001
Not observed	83 (46%)	18 (10%)	101 (56%)	
Undetermined^b^	10 (5.6%)	26 (15%)	36 (20%)	
Observed	24 (13%)	18 (10%)	42 (23%)	
Echogenicity				<0.001
Not observed	89 (50%)	19 (11%)	108 (60%)	
Undetermined^b^	11 (6.1%)	29 (16%)	40 (22%)	
Observed	17 (9.5%)	14 (7.8%)	31 (17%)	
Overall				<0.001
Abnormal	29 (16%)	27 (15%)	56 (31%)	
Normal	80 (45%)	18 (10%)	98 (55%)	
Undetermined	8 (4.5%)	17 (9.5%)	25 (14%)	
Total, N (%)	117 (65%)	62 (35%)	179 (100%)	
(B) Measurement^c^ median (IQR)^d^				
TT3 (nmol/L)	0.59 (0.29, 1.11)	0.31 (0.16, 0.91)	0.55 (0.22, 1.02)	0.089
TT4 (nmol/L)	0.85 (0.10, 2.56)	0.56 (0.01, 2.46)	0.80 (0.04, 2.49)	0.5
FT4 (pmol/L)	0.86 (0.00, 2.02)	0.00 (0.00, 1.32)	0.62 (0.00, 1.86)	0.2
Inorganic iodine (ng/ml)	35.0 (21.0, 58.0)	376.0 (129.0, 1883.0)	48.0 (25.0, 128.0)	<0.001
Total iodine (ng/ml)	79.0 (48.0, 185.0)	962.0 (626.0, 2087.0)	150.0 (53.0, 492.0)	N/A
Cobalt (ng/ml)	88.0 (23.0, 160.0)	388.0 (129.0, 865.0)	112.0 (28.0, 312.0)	<0.001

Ultrasound scores were compared by grouping blood samples into <400 and > 400 ng/mL total iodine, where 400 ng/mL was used as the upper cutoff value in normal, wild SRs. Pearson’s chi-squared test was used to determine differences between the expected and observed frequencies in observations between the two total iodine level groups. (B) Thyroid, iodine and cobalt median and interquartile range (IQR) for the <400 and > 400 ng/mL total iodine groups outlined in A.

a Pearson’s Chi-squared test.

b Undetermined or unknown due to absence of ultrasound or quality sufficient to evaluate.

c Total triiodothyronine (TT3; nmol/L); Total tetraiodothyronine (TT4; nmol/L); Free thyroxine (FT4; pmol/L).

d Interquartile range (IQR) reported here as: (25th, 75th percentile).

e Wilcoxon rank sum test.

Challenges with ultrasound evaluation in these cases included a lack of images from examinations, misinterpretation of anatomy, and inability of the ultrasonographer to obtain good quality images (depth, type of transducer, and optimal adjustments of settings).

#### 3.4.2 Serum total iodine measures <400 and > 400 ng/mL as a proxy for vitamin/mineral consumption

Since consumption of the supplement varied by species, individual, and time, and was not directly measured, serum total iodine measurements were used to identify animals with likely consistent consumption (animals that consumed their supplement had higher levels than those that did not). This placed their ranges well above values measured in the unsupplemented wild SR (evaluated as having normal thyroids). Using that information, we parsed the dataset using a normal total iodine cutoff of 400 ng/mL and used the two groups (< or > 400 ng/mL total iodine) to compare the relative proportions of samples with or without cysts or abnormal ultrasound echogenicity, and overall thyroid evaluation (normal vs. abnormal, see Table 3A), as well as a comparison of the relative distribution of thyroid evaluation scores, and their thyroid, cobalt, and inorganic iodine values (see Table 3).

Considering all species and habitats, cysts and echogenicity were less likely to be observed in ultrasounds within the <400 ng/mL total iodine group (46% vs. 10%; and 50% vs. 11%, respectively), and a higher percent of observations were scored as normal in the overall thyroid evaluation (45% vs. 10%; see Table 3A). Samples from animals with serum total iodine >400 ng/mL (*N* = 62) had higher rates of observed cysts [18/62 (29%)] and echogenicity [14/62 (22.6%)] compared to the <400 group.

[24/117 (20.5%)] and [17/117 (14.5%), respectively; see Table 3A]. In the aquarium cohort, fewer thyroid ultrasound scans were evaluated as normal in the >400 total iodine group [12/47 (26%)], and a higher percentage of moderate to severe thyroid evaluation scores, were in the >400 total iodine group [13/18 (72%)]. While there were no significant differences in TT3, TT4, or FT4 between the <400 and > 400 total iodine groups (see Table 3B), as expected, inorganic iodine and cobalt were also higher in the >400 group [Table 3B; I (I) <400I(T) χ̃=35.0, IQR = 21.0, 58.0, vs. I (I) > 400I(T) χ̃=376.0, IQR = 129.0, 1,883.0; *p* < 0.001; and Cobalt <400I(T) χ̃=88.0, IQR = 23.0, 160, vs. Cobalt >400I(T) χ̃=388.0, IQR = 129.0, 365.0; *p* < 0.001].

### 3.5 Thyroid, iodine and cobalt measures as predictors of thyroid disease in the aquarium-housed SR

Ordinal regression models were determined to be the best fit to evaluate the ability of serum inorganic iodine, total iodine (or total iodine <400 or > 400 ng/mL), cobalt, TT3, TT4, and FT4 to predict thyroid disease evaluation scores in the aquarium cohort. In the first model, iodine values were log-transformed, and total iodine and cobalt were removed due to high VIF, collinearity and best fit. For Model 1 the factors determined to best predict thyroid disease severity (Y) was Y ~ log inorganic iodine + TT3 + TT4 + FT4 (Nagelkerke R^2^ = 0.98; see Supplementary Table S3). Holding all other predictors constant, samples with 1 unit higher value of log inorganic iodine were 2.7 times more likely to be evaluated in a higher thyroid evaluation score (OR = 2.738, 95% CI = 1.280, 6.833).

**Figure 7:**
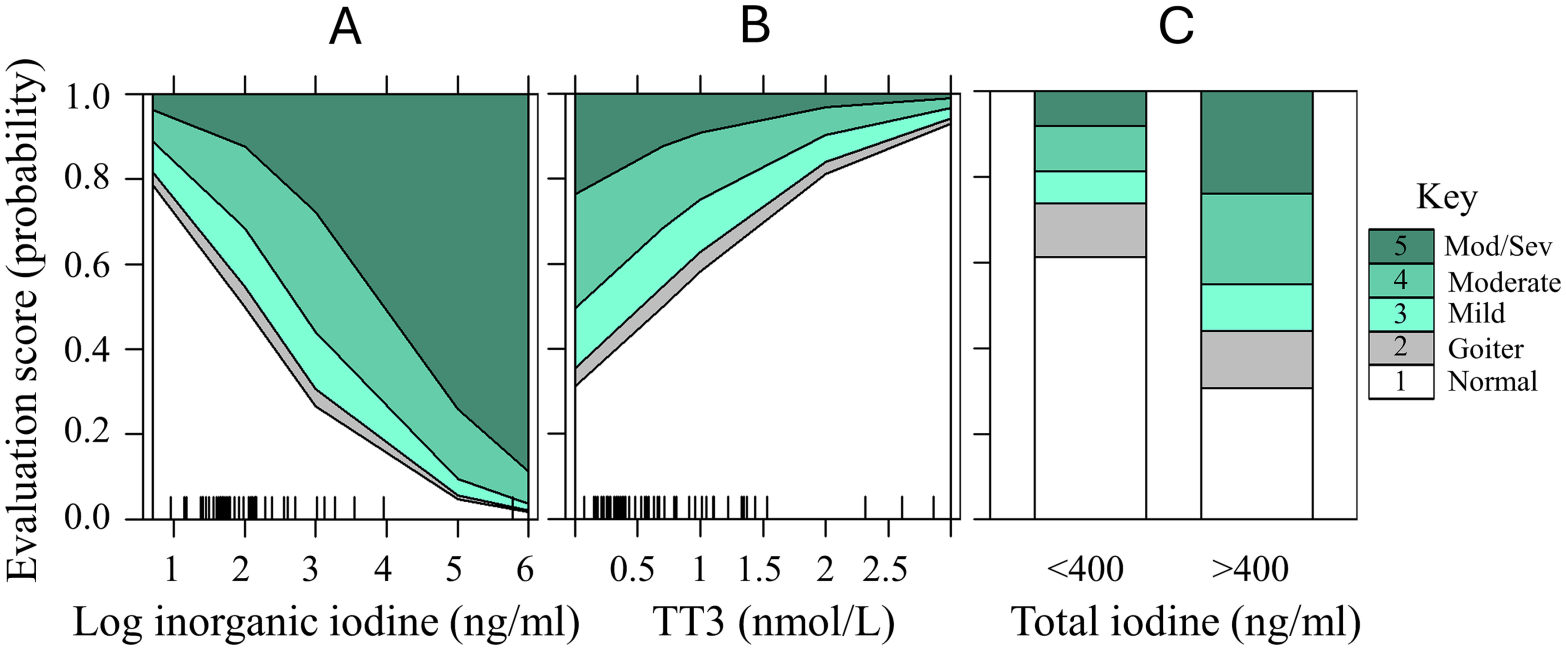
Predictor effects plots with cumulative probability estimates for increasing severity in elasmobranch thyroid evaluation disease (scores 1 through 5; normal through moderate/severe) for predictor variables in ordinal regression Model 1: **(A)** log inorganic iodine (ng/ml); **(B)** TT3 (nmol/L); and for Model 2: **(C)** Total iodine <400 and > 400 ng/mL. Rug plots on the x-axes represent the marginal distribution of the numeric predictors (panels **A,B**). Data from aquarium-managed Study 2 cohort only.

Serum TT3 had a more complex association with iodine and thyroid disease score. Serum TT3 values were elevated in many of the samples from supplemented animals with the exception of the highest severity of thyroid disease (see Figure 6A). This negative relationship can also be seen in Figure 7B predictor effects plot as a negative association of TT3 with thyroid disease such that the highest probability of moderate–severe disease was associated with the lowest TT3 values. Holding all other predictor values constant, with every one nmol/L increase in TT3 we would expect a 1.125 decrease in the cumulative probability of thyroid disease evaluation level (log odds scale). Calculating for the OR (OR = 0.325, 95% CI = 0.091, 0.944) the reciprocal (inverse of the odds ratio = 3.077) can be more easily interpreted. In this model, where increased inorganic iodine was correlated with increasing disease severity, there was also a 3.1 times higher odds to be evaluated into a higher thyroid disease category for every 1 nmol/L decrease observed in serum TT3. The cumulative probabilities for each change in thyroid evaluation score are visualized as predictor effects plots in Figures 7A,B (log inorganic iodine, and TT3).

For Model 2 we were specifically interested in the predictive ability of serum total iodine measures <400 or > 400 ng/mL as categorical variables. Using a final model of Y ~ total iodine >400 + TT3 + TT4 + FT4, samples with total iodine >400 ng/mL were 3.6 times more likely to be evaluated as a higher thyroid disease score (OR = 3.591, 95% CI = 1.280, 6.833, *p* = 0.012). Serum TT3 was not significant in this more generalized model (*p* = 0.104), but had similar trends as in Model 1, with lower TT3 values occurring in the highest disease categories. The differences between the highest thyroid disease evaluations for mild/moderate (*p* < 0.01) and moderate/moderate–severe (*p* < 0.001) can be readily distinguished between the <400 and > 400 ng/mL total iodine categories (Figure 7C). In the <400 ng/mL total iodine category, the probability of a normal thyroid evaluation was 61% (vs. 31% for >400), and only 19% probability of scoring a 4 or 5 (moderate to moderate–severe) in the <400 ng/mL total iodine (vs. 45% probability for >400).

## 4 Discussion

Thyroid disease is a common concern with elasmobranchs, with hyperplastic or diffuse colloidal (“classic”) goiter being the foremost diagnosis. If identified early, morbidity and possible mortality can be prevented. The etiology is frequently linked to iodine, usually as a deficiency originating in low iodine consumption. This occurs because it is unavailable to the animal either from low availability in food or water, ozone treatments of the water, other goitrogenic causes (high nitrates), or a mix of the three (49–53). Diagnosis is made by an obvious enlargement under the jaw. Short of that observation, few studies have shown other methods for diagnosis. Thyroid hormone values have been assessed and reported to be low, when compared to other vertebrates, for both T3 and T4 in goitered sharks (37, 54). As such, thyroid hormone assessment seemed a reasonable test in managed care animals. Interestingly, in the aquarium-managed cohort of animals presented here, the RIA-derived values using standard methodology and sample volumes routinely registered as low or unreadable, even in apparently healthy sharks and rays. There was only one commercial lab with an RIA thyroid assay panel at the time of testing (primarily for canine and feline), prompting a closer investigation of the methodology, assay sensitivity, and testing for validation in elasmobranch species.

### 4.1 Is the current methodology for blood thyroid hormone assessment appropriate for elasmobranchs?

Commercial laboratory analysis of blood thyroid hormone results consistently showed values in elasmobranchs were markedly below those routinely measured in other non-wildlife, terrestrial vertebrate species at MSU VDL (e.g., *Felis catus* or *Canis familiaris*). This was especially unusual given that clinical cases of thyroid disease (not goiter) still resulted in near undetectable blood thyroid hormone levels. Working with the laboratory (Bousianny), extant RIA methodology was reviewed. First, a direct measurement for known thyroid hormone was necessary. As a biological validation, HPLC performed on homogenized and extracted thyroid tissue showed high concentrations of immunoreactive TT3, TT4 and FT4 (Figure 1), while values from a matched cardiac blood sample were low. These results suggest that thyroid hormones are indeed highly conserved in elasmobranchs (55), and exhibited expected cross reactivity with established commercial RIA antisera. Secondly, serum was evaluated from a subset of ‘thyroid-normal’ SR and compared to thyroid abnormal SR. Normal values were uniformly quite low, most at or near zero, and in fact, below the readable range of the kit standard curve. Increased sample volume (2x for TT3 and 8x for TT4) and additional standards (down to the assay lower limit of sensitivity) allowed for improved sample measurement (Tables 2, 3). While both T3 and T4 are found in the thyroid itself, presumably large amounts are not routinely released into circulation resulting in relatively low levels which may not correlate with hypothyroidism.

RIA is generally considered the “gold standard” for thyroid hormone assays. Developed decades before the more current EIAs and CLIAs, RIAs generally had the advantage of improved discrimination and greater sensitivity and could easily be adapted “in-house” to meet the needs of species-specific lower ranges by adjusting working dilutions of the radio-labled tracer or antisera titer (56, 57). In contrast, current commercial labs typically follow a standardized RIA kit protocol using unextracted serum that had been optimized for species with higher thyroid hormone ranges (e.g., canine, feline (21)). Previously published data as well as older research were focused on RIA and likely varied methodology. Many labs may switch to non-RIA assays because of ease and safety (no need for radiation) but since they are often validated for targeted species, they might not be sufficiently sensitive to work well on elasmobranchs. Direct comparison of this study to previously published elasmobranch values is not a simple task. Current commercial labs and assays cannot easily perform historical sample processing or match assay methods (49, 51, 56–61). A further question was which of these values have biologic relevance. Thyroid hormone physiology is reviewed elsewhere (62) but salient content notes that in many species T3 is the biologically active form with T4 being a prohormone that requires conversion (62). Additionally T3 may be produced peripherally in multiple organs, like the liver (in dogfish (*Squalus acanthias*)) and possibly the kidney. Overall, the literature continues to be data-poor for thyroid function in elasmobranchs. What exists is challenging to interpret due to a variety of sample processing and assay methodology, and a potential confound of increased TT3 and TT4 in supplemented animals that may also vary in rates of consumption.

Testing methods for TT4 were changed during the timeframe of this research from RIA to CLIA due to assay kit availability. To examine the usefulness of this new test in comparison to the validated RIA, comparisons were conducted on the same samples, where available (*N* = 54). No correlation was found between the two methods (r^2^ = −0.015). Additionally, CLIA values did not repeat well with the same samples when resubmitted. It is unclear why the repeat samples were not consistent but antisera differences or interference factors within the sample matrix are suspected (6, 63, 64).

These findings overall suggest that only TT4 and TT3 of the current RIA assay have potential value with the assay modifications as described. Using the assay ‘as is’, TT4 and TT3 (without increased sample volume) low values near zero may not mean hypothyroidism, and values that are readable (e.g., elevated TT4 and FT4), may indicate a problem. When disease progressed to a severe category, TT3 concentrations dropped to near baseline. TT4 CLIA results were questionable, and further research should be conducted to validate it.

### 4.2 Is serum iodine a good proxy for thyroid health?

Considering that iodide deficiency is the most likely cause of thyroid enlargement and at the time when this study was initiated, with near zero thyroid hormone results in nearly all samples, serum iodine seemed a plausible tool to explore for deficiency and as a proxy potentially for goiters. There are well established connections between acute iodide loads and thyroid inhibition known as the Wolff-Chaikoff effect (65). Total iodine (including unbound and protein-bound forms) and inorganic iodine (unbound only) were run on serum to establish some baselines and also to see if there were changes related to disease. Wild SRs (considered normal) were compared to semi-wild lagoon-housed SR (natural sea water but nutritionally managed) and closed system aquarium-housed SR, that was overrepresented with reproductive disease. In general, serum iodine levels of <20 ng/mL are abnormally low and 20–40 ng/mL questionably low, while 40–400 ng/mL seems to be a range consistent with a healthy animal. The wild SR stood out with iodine values in the 300 ng/mL range and this is likely because of a diet rich in natural iodine [crustaceans, seaweed, etc. (66, 67)]. Conversely, lagoon-housed SR did not have access to high volumes of natural foods and were fed a managed diet while living in natural sea water. Their diet however differed from aquarium-housed SR in that they did not receive a supplement and only a gel diet offered with low levels of iodine and cobalt.

High iodine values (>400 ng/mL) were frequently identified and associated with a negative health status and abnormal thyroid appearance. In practice, the authors recommend any serum values above 200 ng/mL prompt an evaluation of the animal and its history, thyroid appearance, and any comorbidities. A possible outcome might be a reduction of supplement in a mg/kg of fish or a reduction of frequency per week. Extant supplements that a majority of aquaria use have a moderate amount of iodide in their formulations although this has decreased over the last several years. Others have suggested that the typical level of iodide offered to elasmobranchs is more than required to balance a deficient diet (37, 68).

Serum total and inorganic iodine values in wild SR ranged from 177 to 307 ng/mL, and 32 to 48 ng/mL (respectively). In the lagoon-housed SR, values were lower than expected. These animals were exposed to natural iodide in natural sea water and minor amounts in the diet, but they were not supplemented. Neither of these groups had any thyroid abnormalities identified on physical exam or ultrasound. In the overall dataset including aquarium held animals, there were some very high values of iodine (Figure 2). Levels of supplement correlated with serum levels: if there were higher levels of dietary iodine, this tended to correlate with greater serum iodine levels. Animals that reliably accepted and consumed supplements were documented with elevated iodine levels compared to individuals that did not (pers experience Mylniczenko). One cohort had reproductive disease and abnormally high levels of iodine (>2000 ng/mL, unpublished) (22). Those individuals had all supplementation removed (oral and water sources) for more than a year to observe serum levels of iodine over time as well as thyroid size and shape. Iodine levels dropped but took several weeks to months, suggesting accumulation and slow excretion rate (69), yet the thyroid never enlarged although showed abnormal architecture. This prompted a smaller investigation in healthy animals given one dose of supplement (70) that showed iodine peaked at 24 h and declined over 72 h. This suggests that repeated dosing at an interval less than 72 h may result in accumulation, helping at least in part to explain higher levels of iodine.

This finding highlighted that accumulated iodine levels take a long period of time to reduce and even with seemingly low levels, not all species develop goiters. In contrast, eagle rays not receiving supplements developed a goiter by 6 months post birth. Similarly, bamboo sharks developed goiters 2 months after removal of supplements (52). The water systems they were in were ozonated, making iodide absorption and use from water improbable (50). This is likely due to the assumption that any iodide that comes in contact with ozonated water, potentially even within the digestive tract, would react to become biologically unavailable in the form of iodate (50, 71).

It is tempting to recommend normal ranges or exact cut-offs for low serum iodine to facilitate diagnosis of goiters. However, lagoon-housed SR had both low serum values and low dietary iodine, with only the natural sea water providing biologically available iodine and they did not develop goiters. Iodine can be both absorbed and excreted through renal clearance at a variable rate dependent on species and the presence of other nutrients such as chloride, so the ocean’s bioavailable iodide may be processed differently than in an aquarium setting with faster clearance (12, 72, 73). In the few animals with confirmed goiters, those individuals were not on any supplements prior to diagnosis (pups under observation). Once a goiter was identified, either by gross appearance or ultrasound, supplementation was started and the goiter was resolved.

Hyperiodinism, as shown here, has not really been considered a problem of elasmobranchs. It has been suggested that ‘excess’ waterborne iodide did not change serum T3 and T4 (RIA) concentrations in white tip reef sharks relative to TT3 and TT4 (EIA) when compared to ranges reported in wild-caught Atlantic stingrays (*H. sabina*) (49, 57, 58) and trout (74). This direct comparison is challenged by the variability in delivery method, sample processing, antisera, assay methodology and sensitivity (free vs. total T3 or T4: RIA vs. EIA). The authors suggested no negative consequence from high iodide and concluded that they are insensitive to excess iodide. This is contrary to literature across species which does suggest iodine in excess can cause problems. Excess iodine can lead to thyroid dysfunction resulting in either hyperthyroidism as well as hypothyroidism (65, 75), thyroiditis, and goiter development (76). In some mammals, iodide can cause toxicity resulting in mortality of young animals, prolonged parturition, and other signs (75, 77). In birds, excessive iodine can cause reduced egg production by directly inhibiting ovulation (78, 79). Additionally, at least in fish, the ‘insensitivity’ to iodide was tested in short term high exposure, vs. chronic exposure. In the data presented here, iodine is shown to correlate with consumption, length of time on supplements, and disease state (reproductive disease and abnormal thyroid tissue findings). Some of the levels identified in this paper suggest that iodine may be stored in the body outside of the thyroid gland, allowing for later release, as has been documented in other species (80, 81). Iodine may vary in non-thyroid storage sites across species. It accumulates in bile (80) as well as ova (82) and in some of the animals reported here, follicular iodine was interpreted as high (~9,000–10,000 ng/mL compared to same day serum of >8,000 ng/mL; unpublished) in animals with reproductive disease (characterized by follicular stasis or retention). Normal comparisons are still necessary. In a recent paper on iodinated contrast in catshark (*Scyliorhinus torazame*) computed tomography scans, iodine was seen to accumulate in the gallbladder peaking at day 6 and ovary showing high values at day 260 post administration (83). This storage capacity may help explain some of the high values seen in the current study. It is unclear in males if there is a different level of accumulation as abnormal animals in this study were predominantly female (females comprised 81.7% of individuals & 87.2% of study samples; see Supplementary Table S1). Iodide, in excess, has been labeled as an endocrine disruptor with the thyroid as the main target (84). Additionally, excess iodine has been linked with hyperthyroidism and arrested ovulation which fits with several of the cases here (85). Clearly this is an area of much needed research for this particular disease process (6).

Serum iodine in normal thyroid SR showed a lot of variability with the wild group having higher inorganic iodine relative to the lagoon- and aquarium-housed SR conspecifics. This is likely because of access to iodine rich prey and plant material. In this study, there were not many true goiters to derive any ranges in order to offer diagnostic values for identifying risk of goiter. In fact, the lagoon-housed SR, which were in similar habitats to wild SR (natural sea water environment) had moderately lower iodine values and also showed no evidence of goiter formation evidenced by ultrasonography. These animals do not have access to the diversity of prey species as they are managed in an enclosed lagoon and are fed a prepared diet of shrimp, squid and gel that has a minor amount of iodine present. It is possible that the clearance rate of iodine in the lagoon population is high, but access to biologically available water iodide offers a physiologically appropriate level to prevent goiters.

This current data supports the need to provide supplementation to prevent classic colloidal goiters in elasmobranchs. It also highlights the need to reevaluate the current levels and frequency of recommended supplementation as the physiologic levels of some key elements are elevated over what seems required. While species specific data would be ideal for iodine and trace mineral panels, it seems unlikely that the proposed ranges vary significantly from species to species. Additionally, given the association with both thyroid pathology and reproductive disease in many species further investigation is truly warranted.

Iodine may serve as an indicator for thyroid health, but only for elevated thyroid hormones (‘hyperthyroidism’). Iodine levels in managed animals over 400 ng/mL (the upper limit of what wild animals showed) indicate an abnormal status. We recommend levels above 200 ng/mL prompt a recheck and comparison to thyroid ultrasound, often resulting in a reduction of supplement dose and/or increased interval in between dosing. Perhaps mature females require different levels of supplement. The data here suggests that hypothyroidism cannot be conclusively connected to low thyroid levels or low iodine, without corresponding clinical information (overt swelling or ultrasound). Further attention should be considered for long term effects in aquarium populations.

### 4.3 What is the role of cobalt in elasmobranchs?

Serum cobalt was markedly elevated in aquarium-housed animals compared to wild and lagoon-housed SR counterparts; and higher when total iodine was greater than 400 ng/mL. Comparable studies note values of 1.3–2.6 ng/mL in wild sand tiger sharks (*Carcharias taurus*) (86) and 24.9–180.7 ng/mL in aquarium smooth dogfish (*Mustelus canis*) (87). In the current dataset values ranged from 4.2–2,818.9 ng/mL with the highest belonging to aquarium animals.

Cobalt is known to be stored in the thyroid (88, 89) and in excess has been reported as both causing hypothyroidism (with histologic changes in the thyroid) and having adverse reproductive effects (testicular atrophy and alteration of ovarian function) (90, 91). Cobalt serves as a goitrogen with excessive intake in humans, inhibiting normal iodine uptake by the thyroid, which in turn creates excess iodine, inhibiting thyroid activity (37, 92). Cobalt functions as a systemic radical when unbound by albumin, by generating reactive oxygen species involved with lipid peroxidation (93). In the elasmobranch that does not have albumin, there is potentially increased exposure to the ‘radical’ form (45, 93). We found a range of thyroid disease scores associated with the <300 and > 300 ng/mL cobalt values, including normal thyroid with high serum cobalt. Mild, moderate, and severe thyroid evaluations were almost two times more frequent when cobalt was >300 ng/mL. Chickens will alter deposition of iodine in yolk in response to feeding of cobalt supplementation (46), supporting cobalt’s potential role in reproductive disease.

Cobalt is primarily used in the folate cycle in vitamin B12, with excess excreted in mammals. Orally, it has low toxicity, with 100-fold margin of safety from minimum requirements for feeding domestic species (47). Cobalt can bind iodine in systems of high iodine saturation (94, 95). Pharmacological use of cobalt impacts race horses, due to its chemical interaction with iodine, inhibiting the thyroid (96). Excess cobalt intake is documented to alter thyroid metabolism, and impact goiter development despite iodine sufficient diets in humans (97).

Cobalt was correlated with total iodine and TT3, and moderately so, with inorganic iodine, TT4 and FT4 (Figure 4), though not statistically significant. Elevated cobalt levels, as with iodine, appear to negatively impact the health of elasmobranchs. Theoretically, abnormally elevated levels of either or both iodine and/or cobalt appear to result in thyroid dysfunction. This in turn would result in a slow progression of ovarian dysfunction culminating in reproductive disease. With a higher incidence of both iodine and cobalt in animals with reproductive disease and thyroid pathology, further investigation should be explored.

### 4.4 Ultrasound is a good method to assess thyroid disease

Ultrasound is a powerful tool to evaluate an organ like the thyroid where it can provide antemortem data on thyroid size and appearance. Subtle lesions can be seen as well as an enlarging gland prior to seeing outward signs of a ventral swelling. Here, ultrasound was used to tease out differences of thyroid disease, as traditional goiter was seen in only a few cases, while other thyroid pathology was seen in many cases. This allowed for some broad categorization to help understand some of the dynamics of thyroid hormone, iodine and cobalt. An in-depth study on ultrasound size, echogenic character and descriptions of lesions would be ideal but not feasible in this study. Still, it is clear that ultrasonographic changes to the thyroid correlated with disease.

The degree of disease was only generally categorized. Further research and characterization may be worthwhile. Hormone and iodine levels were correlated with subjective evaluation of disease. Causality is difficult, but (as evidenced in the range of values observed in the aquarium-housed animals with normal thyroid) it is suspected that high levels of iodine preceded thyroid pathology, and high values for an extended period of time would likely be a factor. However, given the location and appearance of the thyroid, successful ultrasonography can be challenging. Additionally, a high resolution transducer is recommended as lesions (cysts and subtle nodules) can easily be missed if the correct technique and transducer are not used.

What is unknown, is what degree of percent damage must occur to the thyroid tissue before functionality is altered. This is true in other species where it has been stated that “there is no simple correlation between morphologic lesions and resultant clinical manifestations. A multinodular goiter, for example, in one instance may be associated with normal thyroid function, in another with hyperfunction, and yet another with hypofunction” (98). Overall, ultrasound proved to be either a good adjunct to characterization of severity of disease or an indicator of disease.

### 4.5 Relationships of disease, thyroid hormone, iodine and cobalt

In the normal shark or ray, thyroid hormone values are low (TT3 < 3 nmol/L, TT4 < 5 nmol/L, and FT4 < 5 pmol/L; Figure 3). If animals were over supplemented or over consume (as indicated by elevated iodine and cobalt levels), all three thyroid parameters will be elevated. Paradoxically, in the most severe disease cases, TT3 dropped while TT4 and FT4 stayed elevated, which speculatively implies loss of functionality.

When looking at all the data together, high values in TT3, TT4, and FT4 positively correlated with degree of morphologic disease (either in size or thyroid lesions) and iodine with a proportional increase in cobalt until the disease became severe and then TT3 and FT4 dropped. The inorganic iodine and TT3 levels, when high, could ‘predict’ or specifically, be able to distinguish the moderate and moderate–severe disease categories. In the normal animals, uniformly, the iodines were low <300 with low TT4 and a TT3 of 1–3 nmol/L. An important factor is that the amounts of iodine and cobalt in the supplement decreased (by internal request), over several years. Concomitant to that is a general decrease in serum iodine and cobalt in the aquarium population. Serum cobalt was elevated when serum iodine was elevated, and over a short period of time, cobalt was proportionately higher than iodines when the supplement was initially lowered in iodine. Cobalt may take longer to reduce in the body than the iodine in this circumstance. Iodine and cobalt were also highest with females with reproductive disease, it is presumed this is due to iodine and cobalt accumulation in the ova (46, 80, 82). Low cobalt levels were found in animals with traditional goiters and in natural sea water habitats.

Goiters are a well known symptom of thyroid disease in elasmobranchs. Here it is shown that thyroid disease can present not only in enlargement of the gland, but also with lesions similar to that described in humans (8, 9). Thyroid disease was closely associated with reproductive disease in the aquarium study population. High iodine and cobalt levels were also associated with reproductive disease. Anecdotally, in the SR in this study, elevated reproductive steroid hormones concomitantly dropped when iodine levels also decreased (unpublished data). Additionally, it is worth noting that not all elasmobranchs get goiters and there are potentially species-specific susceptibilities (37).

Some changes are reversible. In the cases of classical goiters (e.g., eagle rays, *Aetobatus* spp.,), iodine supplementation indeed reversed the enlargement of the thyroid. In others, mineralizations and large cysts were not resolved over time, likely indicating permanent tissue alteration. Ultrasound would provide an excellent data set if collected over time by a uniform sonographer with high resolution linear transducers.

Limitations of this data set include a limited number of animals within species and disease categories and an inability to monitor and qualify consumption of supplementation directly in all cases. Additionally, there was a lack of a robust series of abnormal biological controls in terms of classic goiter to establish some low ranges of iodine. A future direction can include validation of TT4 CLIA especially at the test’s lower detection limits. Additionally, further research can be considered into the interplay of iodine, cobalt and thyroid hormone in elasmobranchs.

## 5 Conclusion

1. Current available assays for thyroid hormone in elasmobranchs are not appropriate to diagnose hypothyroidism, but may be helpful for hyperthyroidism. CLIA testing for TT4 did not correlate with previous RIA values.
2. Thyroid ultrasound was an excellent method to evaluate the size of the thyroid and identify lesions within the tissue using linear transducers.
3. Thyroid disease in elasmobranchs extends beyond classic goiter development, but needs better characterization.
4. Iodine is a promising test for indicating a problem with the thyroid gland.
5. Iodine accumulates over time in elasmobranchs with overconsumption; interaction with high dietary cobalt may influence accumulation.
6. Animals with reproductive disease also had concurrent high levels of iodine and they are overrepresented in this dataset.
7. Iodine serum levels should be assessed routinely in elasmobranchs.

## Author’s note

There is a historical partnership between Disney and Mazuri PMI Nutrition International (whose supplement is referred to herein) for multiple patented feed products, none of which are involved in this publication.

## Data Availability

The original contributions presented in the study are included in the article/Supplementary material, further inquiries can be directed to the corresponding author.

## References

[1] Sage M. The evolution of thyroidal function in fishes. Am Zool. (1973) 13:899–905. doi: 10.1093/icb/13.3.899

[2] Dodd JM Dodd MHI Duggan RT. Control of reproduction in elasmobranch fishes. In: Rankin JC Pitcher TJ Duggan RT, editors. Control processes in fish physiology. London and Canberra: Croom Helm (1983). p. 221–49.

[3] Anderson WG. Endocrine Systems in Elasmobranchs. In: Shadwick RE Farrell AP Brauner CJ, editors. Physiology of elasmobranch fishes: Internal processes. London: Elsevier Inc., (2015). p. 457–530.

[4] Power DM Llewellyn L Faustino M Nowell MA Björnsson BT Einarsdóttir IE. Thyroid hormones in growth and development of fish. Comp Biochem Physiol Part C Toxicol Pharmacol. (2001) 130:447–59. doi: 10.1016/S1532-0456(01)00271-X, PMID: 11738632

[5] Deal CK Volkoff H. The role of the thyroid Axis in fish. Front Endocrinol. (2020) 11:596585. doi: 10.3389/fendo.2020.596585, PMID: 33240222 PMC7681243

[6] Mylniczenko ND Sumigama S Wyffels JT Wheaton CJ Guttridge TL DiRocco S. Ultrasonographic and hormonal characterization of reproductive health and disease in wild, semiwild, and aquarium-housed southern stingrays (*Hypanus americanus*). Am J Vet Res. (2019) 80:931–42. doi: 10.2460/ajvr.80.10.931, PMID: 31556711

[7] Duarte-Guterman P Navarro-Martín L Trudeau VL. Mechanisms of crosstalk between endocrine systems: regulation of sex steroid hormone synthesis and action by thyroid hormones. Gen Comp Endocrinol. (2014) 203:69–85. doi: 10.1016/j.ygcen.2014.03.015, PMID: 24685768

[8] Crow GL Luer WH Harshbarger JC. Histological assessment of goiters in elasmobranch fishes. J Aquat Anim Health. (2001) 13:1–7. doi: 10.1577/1548-8667(2001)013<0001:HAOGIE>2.0.CO;2

[9] Ostrander GK Cheng KC Wolf JC Wolfe MJ. Shark cartilage, Cancer and the growing threat of pseudoscience. Cancer Res. (2004) 64:8485–91. doi: 10.1158/0008-5472.CAN-04-2260, PMID: 15574750

[10] Garner MM. A retrospective study of disease in elasmobranchs. Vet Pathol. (2013) 50:377–89. doi: 10.1177/0300985813482147, PMID: 23528944

[11] Allain P Berre S Krari N Laine-Cessac P Le Bouil A Barbot N. Use of plasma iodine assay for diagnosing thyroid disorders. J Clin Pathol. (1993) 46:453–5. doi: 10.1136/jcp.46.5.453, PMID: 8320325 PMC501257

[12] Schöne F Zimmermann C Quanz G Richter G Leiterer M. A high dietary iodine increases thyroid iodine stores and iodine concentration in blood serum but has little effect on muscle iodine content in pigs. Meat Sci. (2006) 72:365–72. doi: 10.1016/j.meatsci.2005.08.017, PMID: 22061566

[13] Yu S Wang D Cheng X Zhang Q Wang M. Establishing reference intervals for urine and serum iodine levels: a nationwide multicenter study of a euthyroid Chinese population. Clin Chim Acta. (2020) 502:34–40. doi: 10.1016/j.cca.2019.11.038, PMID: 31846617

[14] Gorbman A Lissitzky S Michel R Roche J. Thyroidal metabolism of iodine in the shark *Scyliorhinus (Scyllium) canicula*. Endocrinology. (1952) 51:311–21. doi: 10.1210/endo-51-4-311, PMID: 13010183

[15] Gorbman A. Some aspects of the comparative biochemistry of iodine utilization and the evolution of thyroidal function. Physiol Rev. (1955) 35:336–46. doi: 10.1152/physrev.1955.35.2.336, PMID: 14384510

[16] Allain P Mauras Y Dougé C Jaunault L Delaporte T Beaugrand C. Determination of iodine and bromine in plasma and urine by inductively coupled plasma mass spectrometry. Analyst. (1990) 115:813–5. doi: 10.1039/AN9901500813, PMID: 2393085

[17] Lopez-Rodriguez MF Cymbaluk NF Epp T Laarveld B Thrasher M Card CE. A field study of serum, colostrum, Milk iodine, and thyroid hormone concentrations in postpartum draft mares and foals. J Equine Vet. (2020) 90:103018. doi: 10.1016/j.jevs.2020.103018, PMID: 32534782

[18] Jin X Jiang P Liu L Jia Q Liu P Meng F. The application of serum iodine in assessing individual iodine status. Clin Endocrinol. (2017) 87:807–14. doi: 10.1111/cen.13421, PMID: 28708323

[19] Taurog A Chaikoff I. On the determination of plasma iodine. J Biol Chem. (1946) 163:313–22. doi: 10.1016/S0021-9258(17)41373-1, PMID: 21023654

[20] Tudoreanu L Vișoiu G Crivineanu V. Reference ranges of minerals, thyroid hormones and TSH in canine blood serum. Bull Univ Agric Sci Vet Med Cluj-Napoca Vet Med. (2015) 72:183–4. doi: 10.15835/buasvmcn-vm:10480

[21] Nachreiner RF Refsal KR Petroff B Brudvig J. Endocrinology reference ranges. Lansing, MI: Michigan State University Veterinary Diagnostic Laboratory (2024). Available from: https://cvm.msu.edu/assets/documents/VDL/Endocrinology-Reference-Ranges.pdf

[22] Mylniczenko ND Wyffels J Penfold LM. Progress in Understanding Reproductive Disease is Southern Stingrays (*Dasyatis americana*). Atlanta, GA: American Association of Zoo Veterinarians (2016).

[23] Williams SM Sullivan KE Livingston S Valdes EV Mylniczenko ND. Short term impact of a single dose of iodine supplementation on serum iodine in black blotched stingrays (*Taeniura meyeni*). In: Brooks M Freel T Koutsos E, editors. Proceedings of the Thirteenth Conference on Zoo and Wildlife Nutrition, Zoo and Wildlife Nutrition Foundation and AZA Nutrition Advisory Group. St. Louis, MO. (2019).

[24] Meadows MH. Examining spatial and trophic ecology of Bahamian stingrays, *Styracura schmardae* and *Hypanus americanus*, using stable isotope analysis [Doctoral dissertation]. [United Kingdom]: University of Exeter; (2018).

[25] Tagawa M Hirano T. Changes in tissue and blood concentrations of thyroid hormones in developing chum salmon. Gen Comp Endocrinol. (1989) 76:437–43. doi: 10.1016/0016-6480(89)90140-8, PMID: 2583473

[26] Tagawa M Hirano T. Presence of thyroxine in eggs and changes in its content during early development of chum salmon, (*Oncorhynchus keta*). Gen Comp Endocrinol. (1987) 68:129–35. doi: 10.1016/0016-6480(87)90068-2, PMID: 3666420

[27] Leatherland JF Lin L Down NE Donaldson EM. Thyroid hormone content of eggs and early developmental stages of five *Oncorhynchus* species. Can J Fish Aquat Sci. (1989) 46:2140–5. doi: 10.1139/f89-264

[28] Sweeting RM Eales JG. HPLC analysis of in vitro hepatic deiodination products of thyroid hormones in the rainbow trout, (*Oncorhynchus mykiss*). Gen Comp Endocrinol. (1992) 85:367–75. doi: 10.1016/0016-6480(92)90081-T, PMID: 1577240

[29] Gika H Lämmerhofer M Papadoyannis I Lindner W. Direct separation and quantitative analysis of thyroxine and triiodothyronine enantiomers in pharmaceuticals by high-performance liquid chromatography. J Chromatogr B. (2004) 800:193–201. doi: 10.1016/j.jchromb.2003.07.005, PMID: 14698255

[30] Wasser SK Azkarate JC Booth RK Hayward L Hunt K Ayres K. Non-invasive measurement of thyroid hormone in feces of a diverse array of avian and mammalian species. Gen Comp Endocrinol. (2010) 168:1–7. doi: 10.1016/j.ygcen.2010.04.004, PMID: 20412809

[31] Wheaton CJ Mylniczenko ND Rimoldi JM Gadepalli RSVS Hart R O’Hara BR. Challenges, pitfalls and surprises: development and validation of a monoclonal antibody for enzyme immunoassay of the steroid 1α-hydroxycorticosterone in elasmobranch species. Gen Comp Endocrinol. (2018) 265:83–9. doi: 10.1016/j.ygcen.2018.01.028, PMID: 29409969 PMC6068012

[32] Wahlen R Evans L Turner J Hearn R. The use of collision/reaction cell ICP-MS for the determination of elements in blood and serum samples. (2005) 20:84–9.

[33] Mylniczenko ND Culpepper EE Clauss T. Diagnostic imaging of elasmobranchs: updates and case examples. In: The elasmobranch husbandry manual II. Columbus, Ohio: Ohio Biological Survey, Inc. (2017). p. 303–24.

[34] Volkoff H. The thyroid gland of elasmobranch fishes: Structure, function and relations to reproduction and development [Ph. D. Thesis]. Clemson University; (1996).

[35] Richman DM Frates MC. Ultrasound of the Normal thyroid with technical pearls and pitfalls. Radiol Clin North Am. (2020) 58:1033–9. doi: 10.1016/j.rcl.2020.06.006, PMID: 33040846

[36] Lee MK Na DG Joo L Lee JY Ha EJ Kim JH. Standardized imaging and reporting for thyroid ultrasound: Korean Society of Thyroid Radiology Consensus Statement and Recommendation. Korean J Radiol. (2023) 24:22–30. doi: 10.3348/kjr.2022.0894, PMID: 36606617 PMC9830140

[37] Crow GL. Goiter in elasmobranchs. In: Smith M Warmolts D Thoney D Hueter R, editors. The elasmobranch husbandry manual: Captive care of sharks, rays and their relatives. Columbus, Ohio: Ohio Biological Survey, Inc. (2004). p. 441–6.

[38] R Core Team. R: A language and environment for statistical computing. Vienna, Austria: R Foundation for Statistical Computing (2023).

[39] Wood M. Statistical inference using bootstrap confidence intervals. Significance. (2004) 1:180–2. doi: 10.1111/j.1740-9713.2004.00067.x

[40] Wei T Simko V. “R package ‘corrplot’: visualization of a correlation matrix.” Version 0.92. (2021). Available at: https://github.com/taiyun/corrplot

[41] Harrell Jr FE. Harrell Miscellaneous [R package Hmisc version 5.1-1]. Comprehensive R Archive Network (CRAN). (2023). Available at: https://CRAN.R-project.org/package=Hmisc

[42] Friendly M. Corrgrams: exploratory displays for correlation matrices. Am Stat. (2002) 56:316–24. doi: 10.1198/000313002533

[43] Fox J Hong J. Effect displays in *R* for multinomial and proportional-odds logit models: extensions to the effects package. J Stat Softw. (2009) 32:1–24. doi: 10.18637/jss.v032.i01

[44] Fox J Weisberg S. Visualizing fit and lack of fit in complex regression models with predictor effect plots and partial residuals. J Stat Softw. (2018) 87:1–27. doi: 10.18637/jss.v087.i09

[45] Ćwiertnia A Kozłowski M Cymbaluk-Płoska A. The role of Iron and cobalt in gynecological diseases. Cells. (2022) 12:117. doi: 10.3390/cells12010117, PMID: 36611913 PMC9818544

[46] Aghdashi MN Nobakht A Mehmannavaz Y. Laying hen performance and yolk iodine, cobalt and iron content increased by dietary supplementation of potassium iodide. Vitamin B12 and Ferrous Sulfate. (2021). PREPRINT (Version 1). Available at Research Square [10.21203/rs.3.rs-485188/v1].

[47] Suttle N. Copper In: Mineral nutrition of livestock. 5th ed. GB: CABI (2022). 259–300.

[48] Bojunga J. Ultrasound of thyroid nodules. Ultraschall Med-Eur J Ultrasound. (2018) 39:488–511. doi: 10.1055/a-0659-2350 30176696

[49] Crow GL Atkinson MJ Ron B Atkinson S Skillman ADK Wong GTF. Relationship of water chemistry to serum thyroid hormones in captive sharks with Goitres. Aquat Geochem. (1998) 4:469–80. doi: 10.1023/A:1009600818580

[50] Sherrill J Whitaker BR Wong GTF. Effects of ozonation on the speciation of dissolved iodine in artificial seawater. J Zoo Wildl Med. (2004) 35:347–55. doi: 10.1638/03-025, PMID: 15526890

[51] Morris AL Hamlin HJ Francis-Floyd R Sheppard BJ Guillette LJ. Nitrate-induced goiter in captive Whitespotted bamboo sharks *Chiloscyllium plagiosum*. J Aquat Anim Health. (2011) 23:92–9. doi: 10.1080/08997659.2011.574079, PMID: 21834332

[52] Morris AL Stremme DW Sheppard BJ Walsh MT Farina LL Francis-Floyd R. The onset of goiter in several species of sharks following the addition of ozone to a touch pool. J Zoo Wildl Med. (2012) 43:621–4. doi: 10.1638/2010-0160R2.1, PMID: 23082528

[53] Parkinson L Noblitt SD Campbell T Sladky K. Comparison of two iodine quantification methods in an artificial seawater system housing white-spotted bamboo sharks (*Chiloscyllium plagiosum*). J Zoo Wildl Med. (2018) 49:952–8. doi: 10.1638/2017-0005.1, PMID: 30592916

[54] Stoskopf MK. Fish medicine. Philadelphia, PA: W.B. Saunders Co. (1993) p. 882.

[55] Leatherland JF. Reflections on the thyroidology of fishes: from molecules to humankind. Guelph Ichthyol Rev. (1994) 2:1–67.

[56] Brown S Eales J. Measurement of L-thyroxine and 3, 5, 3′-triiodo-L-thyronine levels in fish plasma by radioimmunoassay. Can J Zool. (1977) 55:293–9. doi: 10.1139/z77-039, PMID: 837287

[57] Crow GL Ron B Atkinson S Rasmussen LEL. Serum T4 and serum T3 concentrations in immature captive whitetip reef sharks. *Triaenodon obesus* J Exp Zool. (1999) 284:500–4. doi: 10.1002/(SICI)1097-010X(19991001)284:5<500::AID-JEZ5>3.0.CO;2-J, PMID: 10469987

[58] Volkoff H Wourms JP Amesbury E Snelson FF. Structure of the thyroid gland, serum thyroid hormones, and the reproductive cycle of the Atlantic stingray. *Dasyatis sabina* J Exp Zool. (1999) 284:505–16. doi: 10.1002/(SICI)1097-010X(19991001)284:5<505::AID-JEZ6>3.0.CO;2-1, PMID: 10469988

[59] Gash TA. Seasonal thyroid activity in the bonnethead shark, *Sphyrna tiburo* [Doctoral dissertation/PhD diss]. Texas A& M University; (2000).

[60] McComb DM Gelsleichter J Manire CA Brinn R Brown CL. Comparative thyroid hormone concentration in maternal serum and yolk of the bonnethead shark (*Sphyrna tiburo*) from two sites along the coast of Florida. Gen Comp Endocrinol. (2005) 144:167–73. doi: 10.1016/j.ygcen.2005.05.005, PMID: 16024019

[61] Rangel BDS Moreira RG Niella YV Sulikowski JA Hammerschlag N. Metabolic and nutritional condition of juvenile tiger sharks exposed to regional differences in coastal urbanization. Sci Total Environ. (2021) 780:146548. doi: 10.1016/j.scitotenv.2021.146548, PMID: 34030348

[62] Leary SC Ballantyne JS Leatherland JF. Evaluation of thyroid hormone economy in elasmobranch fishes, with measurements of hepatic 5′-monodeiodinase activity in wild dogfish. J Exp Zool. (1999) 284:492–9. doi: 10.1002/(SICI)1097-010X(19991001)284:5<492::AID-JEZ4>3.0.CO;2-A, PMID: 10469986

[63] Martin B. Steroid-protein interactions in nonmammalian vertebrates. Gen Comp Endocrinol. (1975) 25:42–51. doi: 10.1016/0016-6480(75)90037-4, PMID: 235485

[64] Selby C. Interference in immunoassay. Ann Clin Biochem Int J Lab Med. (1999) 36:704–21. doi: 10.1177/000456329903600603, PMID: 10586307

[65] Leung AM Braverman LE. Consequences of excess iodine. Nat Rev Endocrinol. (2014) 10:136–42. doi: 10.1038/nrendo.2013.251, PMID: 24342882 PMC3976240

[66] Sprague M Chau TC Givens DI. Iodine content of wild and farmed seafood and its estimated contribution to UK dietary iodine intake. Nutrients. (2021) 14:195. doi: 10.3390/nu14010195, PMID: 35011067 PMC8747335

[67] Roseland JM Spungen JH Patterson KY Ershow AG Gahche JJ Pehrsson PR. USDA, FDA, and ODS-NIH Database for the Iodine Content of Common Foods [Internet]. (2022). Available from: https://www.ars.usda.gov/ARSUSERFILES/80400535/DATA/IODINE/IODINE%20DATABASE_RELEASE_2_DOCUMENTATION.PDF

[68] Stoskopf M. Shark diagnostics and therapeutics: A short review. J. Aquac. Aquat. Sci. (1990) 5:33–43.

[69] Williams SM Ardente AJ Mylniczenko ND Guttridge TL Sullivan KM Livingston S. Impact of removing dietary supplementation on serum nutrient concentrations in a managed population of southern stingray (Dasyatis americana). In: Proceedings of the Zoo and Wildlife Nutrition Foundation/ AZA Nutrition Advisory Group Twelfth Conference on Zoo and Wildlife Nutrition, September 24-27, Fresno, TX. (2017).

[70] Williams SM Sullivan KE Livingston S Valdes EV Mylniczenko ND. “Short term impact of a single dose of iodine supplementation on serum iodine in black blotched stingrays (*Taeniura meyeni*).” In: *Proceedings of the Thirteenth Conference on Zoo and Wildlife Nutrition, Zoo and Wildlife Nutrition Foundation and AZA Nutrition Advisory Group*. Saint Louis, MO; (2019).

[71] Pulassery S Abraham B Ajikumar N Munnilath A Yoosaf K. Rapid iodine value estimation using a handheld Raman spectrometer for on-site, reagent-free authentication of edible oils. ACS Omega. (2022) 7:9164–71. doi: 10.1021/acsomega.1c05123, PMID: 35350360 PMC8945061

[72] Riggs DS. Quantitative aspects of iodine metabolism in man. Pharmacol Rev. (1954) 6:132–1. 12993583

[73] Zicker S Schoenherr B. Focus on nutrition: the role of iodine in nutrition and metabolism. Compend Contin Educ Vet. (2012) 70:E1–2. doi: 10.1111/j.1753-4887.2012.00541.x 23532759

[74] Eales JG Cyr DG Cook RF. Effect of excess iodide on thyroid function of rainbow trout, *Salmo gairdneri*. Fish Physiol Biochem. (1986) 1:171–7. doi: 10.1007/BF02311133, PMID: 24233117

[75] Bürgi H. Iodine excess. Best Pract Res Clin Endocrinol Metab. (2010) 24:107–15. doi: 10.1016/j.beem.2009.08.010, PMID: 20172475

[76] Pennington JA. A review of iodine toxicity reports. J Am Diet Assoc. (1990) 90:1571–81. doi: 10.1016/S0002-8223(21)01843-5 2229854

[77] Arrington LR Taylor RN Ammerman CB Shirley RL. Effects of excess dietary iodine upon rabbits, hamsters. Rats and Swine J Nutr. (1965) 87:394–8. doi: 10.1093/jn/87.4.394, PMID: 5841855

[78] Preedy VR Burrow GN Watson RR. Comprehensive handbook of iodine: Nutritional, biochemical, pathological, and therapeutic aspects. Amsterdam Boston: Elsevier/Academic Press (2009).

[79] Lewis PD. Responses of domestic fowl to excess iodine: a review. Br J Nutr. (2004) 91:29–39. doi: 10.1079/BJN20031017, PMID: 14748936

[80] Leloup J Fontaine M. Iodine metabolism in lower vertebrates. Ann N Y Acad Sci. (1960) 86:316–53. doi: 10.1111/j.1749-6632.1960.tb42815.x 14415546

[81] Schöne F Rajendram R. Iodine in farm animals. In: Comprehensive Handbook of Iodine [Internet]. Elsevier (2009) 151–70. Available from: https://linkinghub.elsevier.com/retrieve/pii/B9780123741356000169

[82] Mathews DM Johnson NP Sim RG O’Sullivan S Peart JM Hofman PL. Iodine and fertility: do we know enough? Hum Reprod. (2021) 36:265–74. doi: 10.1093/humrep/deaa312, PMID: 33289034

[83] Ito T Furuya M Tanaka T Yoshii Y Murata M Sasai K. Long-term effects of iopamidol as a contrast medium for computed tomography in cloudy catsharks *Scyliorhinus torazame*. J Aquat Anim Health. (2024) 36:239–49. doi: 10.1002/aah.10219, PMID: 38643364

[84] Karbownik-Lewińska M Stępniak J Iwan P Lewiński A. Iodine as a potential endocrine disruptor—a role of oxidative stress. Endocrine. (2022) 78:219–40. doi: 10.1007/s12020-022-03107-7, PMID: 35726078 PMC9584999

[85] Mahapatra D Chandra AK. Biphasic action of iodine in excess at different doses on ovary in adult rats. J Trace Elem Med Biol. (2017) 39:210–20. doi: 10.1016/j.jtemb.2016.10.006, PMID: 27908417

[86] Hoopes LA Clauss T Wetherbee BM Fox DA. Baseline health and nutritional parameters of wild sand tigers sampled in Delaware Bay. J Aquat Anim Health. (2022) 34:101–15. doi: 10.1002/aah.10156, PMID: 35437805 PMC9796768

[87] Persky ME Williams JJ Burks RE Bowman MR Ramer JC Proudfoot JS. Hematologic, plasma biochemistry, and select nutrient values in captive smooth dogfish (*Mustelus canis*). J Zoo Wildl Med. (2012) 43:842–51. doi: 10.1638/2012-0002R1.1, PMID: 23272352

[88] Brewer K Maylin GA Fenger CK Tobin T. Cobalt use and regulation in horseracing: a review. CEP. (2016) 12:1–10. doi: 10.3920/CEP140008

[89] Lantin AC Mallants A Vermeulen J Speybroeck N Hoet P Lison D. Absence of adverse effect on thyroid function and red blood cells in a population of workers exposed to cobalt compounds. Toxicol Lett. (2011) 201:42–6. doi: 10.1016/j.toxlet.2010.12.003, PMID: 21182909

[90] Finley BL Monnot AD Paustenbach DJ Gaffney SH. Derivation of a chronic oral reference dose for cobalt. Regul Toxicol Pharmacol. (2012) 64:491–503. doi: 10.1016/j.yrtph.2012.08.022, PMID: 22982439

[91] Paksy K Olajos F Tóth BE Náray M Tátrai E Huszár L. Uptake and distribution of Cobakt in rat ovaries and pituitary: acute effects of cobalt on Preovulatory luteinizing hormone, follicle stimulating hormone, prolactin and progesterone levels, and on ovulation in rats. Cent Eur J Occup Environ Med. (1999) 5:313–23.

[92] Miller ER Ammerman CB. Iodine bioavailability. In: Bioavailability of nutrients for animals. San Diego CA: Academic Press (1995). 157–67.

[93] Leyssens L Vinck B Van Der Straeten C Wuyts F Maes L. Cobalt toxicity in humans—a review of the potential sources and systemic health effects. Toxicology. (2017) 387:43–56. doi: 10.1016/j.tox.2017.05.015 28572025

[94] Favaudon V Momenteau M Lhoste JM. An unusual cobalt complex. Reaction of cobalt (II) porphyrin with iodine in apolar solvents. Inorg Chem. (1979) 18:2355–7. doi: 10.1021/ic50199a005

[95] Butova VV Bulanova EA Polyakov VA Guda AA Aboraia AM Shapovalov VV. The effect of cobalt content in Zn/co-ZIF-8 on iodine capping properties. Inorg Chim Acta. (2019) 492:18–22. doi: 10.1016/j.ica.2019.04.011

[96] Kinobe RT. Towards the elimination of excessive cobalt supplementation in racing horses: a pharmacological review. Res Vet Sci. (2016) 104:106–12. doi: 10.1016/j.rvsc.2015.12.007, PMID: 26850547

[97] Elizabeth K Mohammed M Devakumar V Fameesh A. Goiter in pre-pubertal children despite urinary iodine sufficiency. J Pediatr Assoc India. (2015) 4:198–201. doi: 10.4103/2667-3592.300847

[98] Robbins SL. Thyroid gland In: Cotran RS Kumar V Robbins SL, editors. Robbins pathologic basis of disease. Philadelphia, PA, USA: W. B. Saunders (1994). 1121–33.

