# Supplementary material for: Investigation of serum thyroid hormones, iodine and cobalt concentrations across common aquarium-housed elasmobranchs
Source: Front Vet Sci. 2025 Feb 26;12:1504527. doi: 10.3389/fvets.2025.1504527 (PMC11924943; doi:10.3389/fvets.2025.1504527)
Supplement: Supplementary file 1 [file Supplementary_file_1.docx]

**Supplementary Materials**

Table S1. Study animals’ habitat, elasmobranch species, size (disk width or total length), sex, blood sample collection years, and number of samples.

| Habitat and species  (N individuals) | Scientific name | Disk width/ total length (cm)^a^ | Sex (M.F) | Sampling years | Sample (N) |
| --- | --- | --- | --- | --- | --- |
| Aquarium-managed (N=38) |  |  | (13.25) |  |  |
| Brownbanded bamboo shark (BBBS) | *Chiloscyllium punctatum* | 66-73 | (0.1) | 2016-2020 | 8 |
| Cownose ray (CNR) | *Rhinoptera bonasus* | 98-117 | (0.4) | 2015-2021 | 7 |
| Honeycomb ray (HCR) | *Himantura undulata* | 44.5-133.5 | (2.4) | 2015-2020 | 32 |
| Porcupine ray (PR) | *Urogymnus asperrimus* | 65-99 | (0.1) | 2016-2020 | 7 |
| Round ribbontail ray (RRR) | *Taeniura meyeni* | 37-184 | (0.3) | 2017-2020 | 13 |
| Spotted eagle ray (SER) | *Aetobatus narinari* | 52-145.5 | (5.4) | 2002-2021 | 29 |
| Southern stingray (SR) | *Hypanus americanus* | 54-112 | (6.5) | 2015-2017 | 13 |
| Whitespotted bamboo shark (WSBS) | *Chiloscyllium plagiosum* | 64-98 | (0.3) | 2014-2021 | 34 |
| Lagoon-managed (N=22) |  |  |  |  | 22 |
| Southern stingray (SR) | *Hypanus americanus* | 73.5-105.5 | (0.22) | 2013-2018 | 25 |
| Wild (N=11) |  |  |  |  | 11 |
| Southern stingray (SR) | *Hypanus americanus* | 54-95 | (0.11) | 2018 | 11 |
| Totals (N=71) | |  | (13.58) | 2002-2021 | 179 |

^a^ For sharks, total length was measured from the tip of the rostrum to the peduncle and then the tip of the tail.

Table S2. Number (N) and percent of total N of each thyroid ultrasound evaluation score by elasmobranch species and habitat (cohort) at the time of blood sampling (N) across species and habitats (aquarium, lagoon or wild). Thyroid ultrasound evaluation score indicated the presence or absence (or undetermined/unknown) of visible cysts, echogenicity, abnormal architecture or relative size.

|  | **Thyroid Ultrasound Evaluation** | | | | | | | **Sample** |
| --- | --- | --- | --- | --- | --- | --- | --- | --- |
| **Habitat & Species (N)** | **Undetermined^a^** | **Normal** | **Normal (Large)** | **Goiter** | **Mild** | **Moderate** | **Moderate/ Severe** | **N**  **(% of total)** |
| **Aquarium** | | | | | | | | |
| Brownbanded bamboo shark (BBBS) | - | 8 | - | - | - | - | - | 8 (4%) |
| Cownose ray (CNR) | - | 2 | - | - | 5 | - | - | 7 (4%) |
| Honeycomb ray (HCR) | 13 | 7 | 5 | - | 3 | - | 4 | 32 (18%) |
| Porcupine ray (PR) | - | - | 7 | - | - | - | - | 7 (4%) |
| Round ribbontail ray (RRR) | 1 | 6 | 2 | - | - | 4 | - | 13 (7%) |
| Spotted eagle ray (SER) | - | 20 | - | 9 | - | - | - | 29 (16%) |
| Southern stingray (SR) | - | 4 | 1 | - | 2 | 6 | - | 13 (7%) |
| Whitespotted bamboo shark (WSBS) | 12 | - | - | - | 5 | 3 | 14 | 34 (19%) |
| **Natural sea water (Lagoon)** | | | | | | | | |
| Southern stingray (SR) | - | 25 | - | - | - | - | - | 25 (14%) |
| **Natural sea water (Wild)** | | | | | | | | |
| Southern stingray (SR) | - | 11 | - | - | - | - | - | 11 (6%) |
| **Evaluation Score N (% of total)** | **9 (5%)** | **83 (46%)** | **15 (8%)** | **26 (14%)** | **15 (8%)** | **13 (7%)** | **18 (10%)** | **179 (100%)** |

^a^ Undetermined or unknown. Ultrasound image not sufficient in quality or view/availability to provide a thyroid evaluation score.

High performance liquid chromatography (HPLC)

Reagents

All reagents used were LC/MS or HPLC analytical grade quality. Purified hormones used for HPLC reference standards or for testing as potential cross reactants (Sigma Aldrich, St. Louis, MO, or Fisher Scientific, Hampton, NH) include: EtOH (Ethanol 200 proof, #459828), MeOH (Methanol, #A452-1), acetonitrile with 0.1% TFA (v/v, Trifluoroacetic Acid (v/v), Optima™ #6000063), T2 (3,3′-Diiodo-L-thyronine, #719536), T3 (3,3′,5-Triiodo-L-thyronine, #T2877), T4 (L-Thyroxine, #T2376), Iodide (certified reference material #41271, 1000 mg/L); and the following certified reference materials for thyroid standards (Cerilliant® 100 μg/mLin Methanol: T-073T4, T-074 T3, and T-075 reverse T3 (rT3)).

As a biological validation, thyroid tissue was retrieved from a wild-caught (bycatch) SR (Hendon) and stored frozen at -80C. Thyroid tissue was thawed, homogenized and 0.25 g was extracted with ice cold MeOH, centrifuged and the supernatant was passed through a syringe filter before HPLC fractionation for RIA of TT3 (total triiodothyronine, nmol/L); TT4 (total thyroxine, nmol/L; FT4 (free thyroxine, pmol/L).

Results from a HPLC chromatogram (including peak retention time (RT) of the Cerilluant thyroid standards and iodide) and the RIA immunoreactivity profile of the thyroid methanolic extract was used to characterize immuno-detectable TT3, TT4 and FT4. Use of methanol-based standards (and sample extractions) in the ACN/1%TFA system produced an exaggerated solvent wave front that comprised minutes 0 to 2.5 to ~3. This may complicate any precise interpretation of eluted peaks. Two standards (I2 and T2) partially co-eluted with the wavefront and had retention times (RT) as follows: iodide (I2) in a wide band at 1-1.83 RT, T2 (2.073-2.683 RT), whereas T3 and T4 separation was more distinct (T3 (2.1 RT (single), 3.78-4.033 RT (panel)), rT3 (3.9 - 4.1 (single), 4.28-4.55 RT (panel)), and T4 (4.18- 4.44 (single), 5.6-6.09 RT (panel)). Homogenized, methanol-extracted thyroid tissue injected onto the column (100ul) produced a very wide band starting at the wave front in fr1-2 (the wavefront) and continuing through fr3 and 4, indicating that 100ul was likely an excess in volume. RIA immunoreactivity in the FBS diluent was low (measuring 0 nmol/L TT3; 1 nmol/L TT4; and 1 pmol/L FT4), and these baseline FBS thyroid values were subtracted from the values from each fraction before analysis. RIA results from thyroid tissue extract fractions indicated that the highest relative percent immunoreactivity in the TT3 assay was found in a wide band starting at the wave front in fractions (fr) 1-2 (the solvent wavefront, not shown) and continued, in part, into fraction 3. An unexpected and unidentified immunoreactivity peak was observed in fractions 2-3 in TT4 and FT4 RIA. Considering fr 4-14 only, a total of 97% of the TT3 immunoreactivtiy was found in fr4, 96% of the TT4 immunoreactivtiy in fr4-7, and 98% of the FT4 immunoreactivtiy in fr4-7. Analysis of rT3 standard showed significant immunoreactivity in the TT4 (>32 nmol/L, 200ul test vol) and FT4 (64 pmol/L, 50ul test vol), but not TT3 assay (0 nmol/L, after accounting for a 1 nmol/L value in the diluent). Conversely, analysis of the T2 standard showed significant immunoreactivity in the TT3 assay (>4.7 nmol/L) but not in the TT4 or FT4 assay (after accounting for a 1 nmol/L value in the diluent). The RT for T3 and rT3 occurs partially during the solvent wavefront through fraction 4, and thus potentially high amounts of native T3 or rT3 in thyroid tissue may have contributed to the initial TT4 and FT4 immunoactivity observed in fr3 (see Figure 1). Cardiac blood collected from the animal at the time of thyroid tissue recovery had low concentrations of TT3 (run at 100 ul volume), TT4 (run at 200 ul volume) and FT4 (0.00 nmol/L, 1.13 nmol/L, and 0.00 pmol/L, respectively), potentially indicating that thyroid hormones available in the thyroid tissue itself were not actively released into circulation, and low thyroid concentrations may not correlate with hypothyroid condition.

(Figure 1 from the main text repeated here for ease of review)


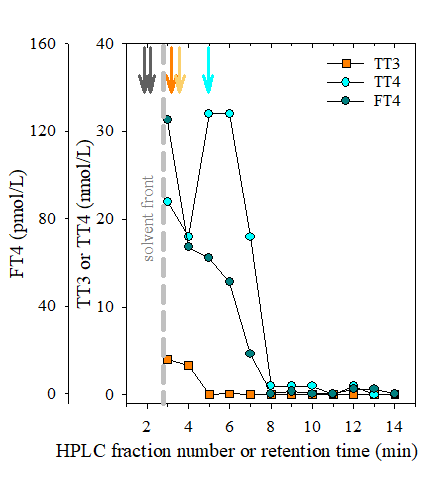


Figure 1 High performance liquid chromatography (HPLC) chromatogram including fraction (fr) number and retention time (RT) peaks from injection of methanolic iodide and thyroid standards (denoted by vertical arrows) or immunoreactivity results from fractions collected following injection of a methanolic extract of thyroid tissue (collected (Hendon) from *H. americanus*) run in commercially available RIAs (radioimmunoassay; TT3 (total triiodothyronine, nmol/L, orange square); TT4 (total thyroxine, nmol/L, cyan circle; FT4 (free thyroxine, pmol/L, dark teal circle). Reference standard RTs are depicted as vertical arrows shown in order from highest to lowest polarity: Iodide and T2 (dark brown), T3 (orange), rT3 (light orange), T4 (cyan). The solvent front is denoted by a vertical gray dashed line.

**Principal Component Analysis (PCA)**

The R packages ‘FactoMineR’ and ‘FactoInvestigate’ were used to explore and visualize Principal Component Analysis (PCA) and Factor Analysis of Mixed Data (FAMD) (1-2) on N = 179 elasmobranch serum samples with 11 variables (Total Iodine, Inorganic Iodine, TT3, TT4 and FT4; using elasmobranch Species (see Table S1 for species abbreviations), Habitat (wild, lagoon- or aquarium-,managed), thyroid disease evaluation code (Evaluation Score: ultrasound evaluations of the thyroid at the time of blood sample collection: normal (wild, lagoon, or aquarium), normal(large), goiter, mild, moderate, or moderate-severe). Iodine supplement factor, Cobalt supplement factor as illustrative qualitative variables. To improve PCA and FAMD, quantitative measures were reduced when two similar measures exhibited a strong correlation (eg. PCA utilized only inorganic iodine in the analysis). Vitamin treatment (yes or no), Iodine and Cobalt factors were given ordinal values based on increased content in the vitamin mineral supplements (eg. 0, 1, 4, 150 for cobalt and 0, 50K(K=1000), 100(K), 200(K) for total iodine; see Table 1 in main text). FAMD and PCA analyses used scaled measures where appropriate. One animal with extremely high Iodine measures was flagged as an outlier and removed from the analysis to prevent disproportionate contribution to the plane of that dimension. To improve PCA and FAMD, quantitative measures were reduced when two similar measures exhibited a strong correlation (see groups representation figure). Investigation of Species as a factor revealed that the effect was primarily due to the (purposeful) presence or absence of thyroid disease (planned comparison groups), vitamin supplementation (iodine and cobalt) that could be pared down to (assumed) relative consumption, that was better indexed by serum total and inorganic iodine values as a proxy. Investigation of initial results (Figures S1 ABC: PCA Correlation circle, FAMD groups representation and ellipse plots by factors of interest (Species; Ultrasound (normal, abnormal, unk/undetermined), Habitat, and thyroid disease score evaluation) were used to drive further analysis of overall relationships between thyroids, iodine and cobalt measures via correlation analyses (section 3.3 and Figure 4. Correlation matrix in main text) and linear and ordinal regression models (see section 3.5 and Figure 7. Predictor effects plots in main text).

Figure S1 A, B, and C Displays principal component analysis (PCA) plots, which shows similarities between groups of samples in a data set. Each arrow (or point) on a PCA plot (S1A) represents a correlation between an initial variable and the first and second principal components (Dimension 1, thyroids (20.19% of the explained variance) on the x axis, and Dimension 2, inorganic iodine (measure) and cobalt and iodine component/level in the supplement (25.89% of the explained variance) on the y axis). Panel S1B displays the barycenter (weighted center of mass of a set of 2D weighted points) for each group (factor). Panel S1C plots each sample by color for each level of each factor (Species; Ultrasound (normal, abnormal, unk/undetermined), Habitat (natural sea water lagoon or wild; or aquarium-managed), and thyroid disease score evaluation. Confidence ellipses drawn around each factor level indicate whether the categories of the supplementary variable are significantly different from each other. In the habitat and thyroid evaluation panels, it can be seen that normal (natural sea water, wild), goiter and moderate-severe thyroid disease group ellipses are the most distinct from the other groups, and SER (spotted eagle ray; *Aetobatus narinari*) and WSBS (white spotted bamboo shark; *Chiloscyllium plagiosum*) species data distinguish themselves from the other species. WSBS data primarily include samples from moderate and moderate-severe thyroid disease evaluations, and SER primarily include normal or goiter thyroid disease evaluations. Most of the other species included a range of normal to diseased, producing overlap in ellipses.

Figure S1A


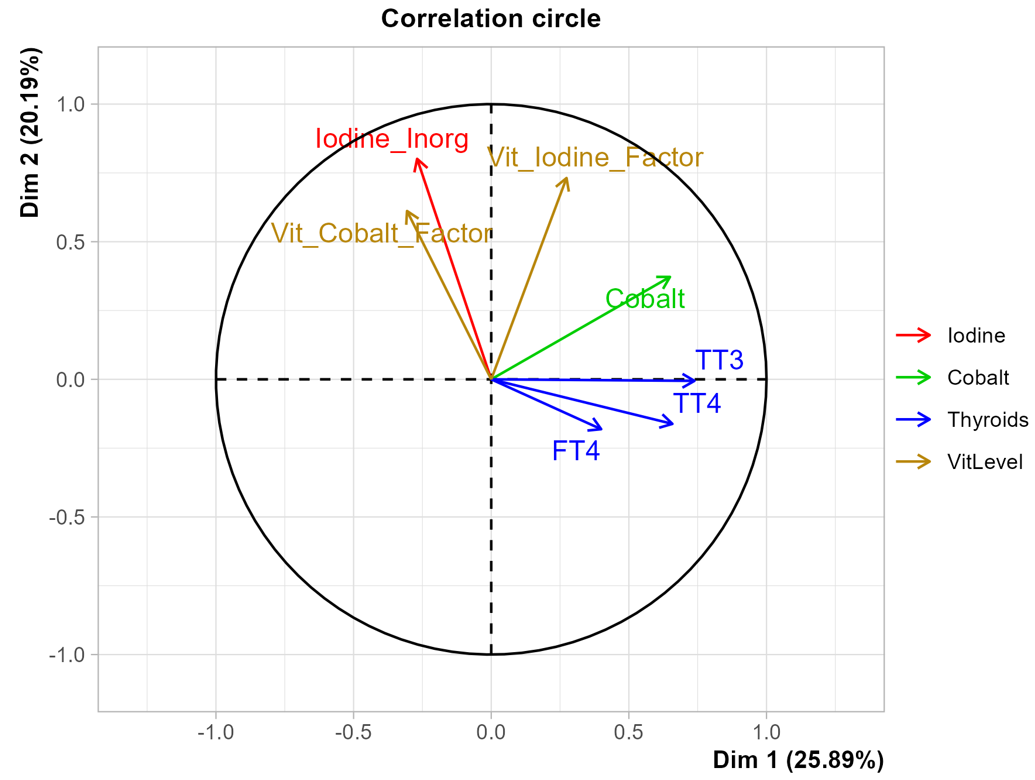


Figure S1B


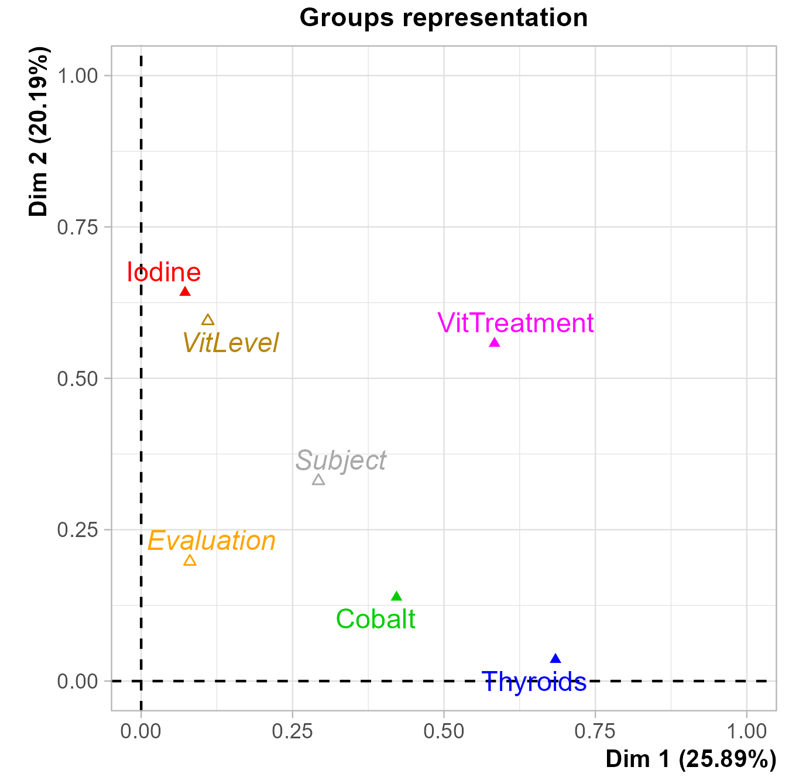


Figure S1C


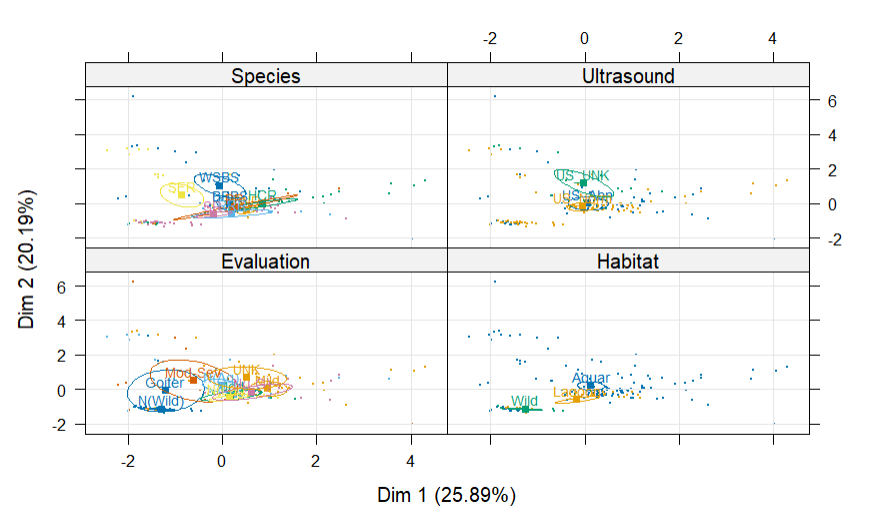


References:

1. Lê S, Josse J, Husson F. FactoMineR : An R Package for Multivariate Analysis. J Stat Soft [Internet]. 2008 [cited 2024 Mar 20];25(1). Available from: <http://www.jstatsoft.org/v25/i01/>
2. Thuleau S, Husson F. FactoInvestigate: Automatic Description of Factorial Analysis. 2023.

Regression analysis of thyroid hormone, iodine and cobalt measures and thyroid disease states (Study 2; Cohort 2)

Methods:

Serum thyroid hormone, iodine and cobalt data from aquarium-managed animals were first visually inspected using the ‘performance’ package (1) to check model assumptions (normality of residuals, normality of random effects, linear relationship, homogeneity of variance, multicollinearity, and VIF (variance inflation factor)). VIF was used to aid in model selection, trimming predictor iodine and thyroid variables where coefficients were inflated due to multicollinearity in the overall model (values with VIF >5 were removed, and modeling re-run).

The associations of thyroid hormone, iodine and cobalt measures with thyroid disease were first investigated using simple linear regression models (lm) using both raw and log-transformed iodine measures with the ‘lme4’ package (2), followed by ordinal regression models. For this modeling, ultrasound evaluations of the thyroid at the time of blood sample collection was transformed into an ordered-factor (normal = 1, goiter = 2, mild = 3, moderate = 4, moderate-severe = 5) for use in multivariable proportional odds logistic regression modeling using ‘MASS::polr’ (3) and ‘ordinal’ (4). Multivariate models were employed testing predictor variables in a ‘drop-one’ manner to determine best model fit. The ‘brant’ package (5) was used to test the underlying assumption of parallelism of the regression. Model selection and overall goodness of fit was evaluated using AIC (Akaike information criterion, (6)), Nagelkerke R2 (proportion of variance 'explained' by the regression model) and log likelihood estimates. Ordinal logit regression modeling was used to evaluate the use of sample iodine (or log-iodine), cobalt and thyroid measures (Model 1; multivariate, continuous data model)). The ‘polr’ function model output provides coefficients given in units of ordered logits, or ordered log odds. For a simpler interpretation of the logistic regression model, we converted each coefficient value into an odds ratio (OR; inverse log of the estimated coefficients). Significant coefficients are reported as odds ratio (OR) and related 95% confidence interval (CI).

Results:

See main text for additional details (section 3.5 Thyroid, iodine and cobalt measures as predictors of thyroid disease in the aquarium-managed cohort). Table S3 displays results for the proportional odds logistic (ordinal) regression model for elasmobranch thyroid disease value (thyroid disease state) including blood sample inorganic iodine, TT3, TT4 and FT4 measures as predictor variables.

Table S3. Results for the proportional odds logistic (ordinal) regression model for elasmobranch thyroid disease value (thyroid disease state) including blood sample inorganic iodine, TT3, TT4 and FT4 measures as predictor variables.

| Ordinal Logistic Regression Model | | | | | | | |
| --- | --- | --- | --- | --- | --- | --- | --- |
| Coefficients^a^ | | | | Analysis of Deviance^b^ | | | Odds Ratio |
| Predictor variable^c^ | Value | SE | Z (*t*-value) | LR Chisq | DF | *P-*  value | OR (95% CI) |
| log Iodine (I) | 1.00706 | 0.42458 | 2.3719 | 6.9874 | 1 | 0.008 ** | 2.738 (1.280, 6.833) |
| TT3 | -1.12504 | 0.58723 | -1.9158 | 4.2965 | 1 | 0.038 * | 0.325 (0.091, 0.944) |
| TT4 | 0.17703 | 0.18095 | 0.9783 | 0.9537 | 1 | 0.329 | 1.194 (0.834, 1.715) |
| FT4 | -0.06279 | 0.07676 | -0.8180 | 0.6611 | 1 | 0.416 | 0.939 (0.805, 1.096) |
| Intercepts | | | | | | | |
| Evaluation | | Value | SE | *t*-value | *P*-value | | |
| Normal/Goiter | | 1.4503 | 1.0278 | 1.4110 | 0.158 | | |
| Goiter/Mild | | 1.6438 | 1.0364 | 1.5860 | 0.113 | | |
| Mild/Moderate | | 2.2271 | 1.0567 | 2.1076 | 0.035* | | |
| Moderate/Mod-Severe | | 3.4206 | 1.1257 | 3.0388 | 0.002** | | |

^a^ Y (eval score) = + 1.01(logIodine(I)) + (-) 1.13(TT3) +(-) 0.06(FT4) + 0.18 (TT4)

^b^ Type II tests

^c^ Total triiodothyronine (TT3); Total tetraiodothyronine (TT4); Free thyroxine (FT4); Inorganic iodine (Iodine (I)).

* p <0.05, ** p < 0.01

References

1. Lüdecke D, Ben-Shachar M, Patil I, Waggoner P, Makowski D. performance: An R Package for Assessment, Comparison and Testing of Statistical Models. J Open Source Softw. 2021 Apr 21;6(60):3139.
2. Bates D, Mächler M, Bolker B, Walker S. Fitting Linear Mixed-Effects Models Using lme4. J Stat Softw [Internet]. 2015 [cited 2023 Apr 27];67(1). Available from: http://www.jstatsoft.org/v67/i01/
3. Venables WN, Ripley BD. Modern applied statistics with S [Internet]. 4th ed. New York: Springer; 2002. Available from: https://www.stats.ox.ac.uk/pub/MASS4/
4. Christensen RHB. ordinal—regression models for ordinal data. R Package Version 202312-4 [Internet]. (2019). Available from: https://CRAN.R-project.org/package=ordinal
5. Schlegel B, Steenbergen M. brant: Test for parallel regression assumption. R Package Version 03-0. 2020.
6. Akaike H. A new look at the statistical model identification. IEEE Trans Autom Control. 1974 Dec;19(6):716–23.
